# Genome-wide association mapping reveals genetic loci underlying phenotypic variation in early root vigour improvement induced by osmopriming in *Brassica napus* L

**DOI:** 10.1186/s12870-025-07540-4

**Published:** 2025-10-29

**Authors:** Kingsley Onyinye Ibeabuchi, Maira Marins Dourado, Stefan Scholten, Ulf Feuerstein

**Affiliations:** 1 Division of Crop Plant Genetics, Department of Crop Sciences, Georg-August-University of Göttingen, Göttingen, Germany; 2 Department of Seed Technology, Deutsche Saatveredelung , Asendorf, Germany; 3 Faculty of Science, Swammerdam Institute for Life Sciences, University of Amsterdam, Amsterdam, Netherlands; 4 Centre for Integrated Breeding Research, Georg-August-University of Göttingen, Göttingen, Germany

**Keywords:** Osmopriming, Polyethylene glycol, Early root vigour, Brassica napus, GWAS, Marker-assisted selection

## Abstract

**Background:**

Seed osmopriming is an effective pre-sowing treatment that enhances early seedling vigour and promotes robust root development in *Brassica napus* L., thereby contributing to improved establishment during the early growth stages. However, the genetic basis of variation in osmopriming responsiveness remains poorly understood. This study employed genome-wide association studies (GWAS) using 15,311 single-nucleotide polymorphisms (SNPs) in 228 spring-type *B. napus* accessions to uncover loci associated with root vigour response to polyethylene glycol (PEG-6000)-mediated osmopriming.

**Results:**

PEG-6000 osmopriming significantly enhanced root vigour traits, increasing average radicle emergence (RE) from 72.7% to 84.4%, root length (RL) by 64.6% (18.2 mm to 29.9 mm), root growth rate (RGR) by 97.8% (1.37 mm/h to 2.71 mm/h), and vigour index (VI) by 88.6% (1355 to 2556) after 72 h of sowing. We also observed substantial phenotypic variation in relative response (e.g., 99.69% CV for RR-VI), which highlights the diversity of osmopriming responsiveness within the population. Our GWAS analysis identified 11 significant SNPs across seven chromosomes (A03, A04, A07, C01, C02, C03, C08), associated with relative responses in four root vigour traits (RR-RE, RR-RL, RR-RGR, and RR-VI). Candidate genes within these loci include regulators of osmotic stress adaptation, water transport, root development, redox balance, and cell wall remodelling. Notably, haplotypes *RR-RE-Hap6* on chromosome A03 and *RR-VI-Hap1* on chromosome C02 were identified as favourable haplotypes for RR-RE and RR-VI, respectively.

**Conclusion:**

Our study provides the first genomic dissection of osmopriming response in *B. napus*, identifying key loci and candidate genes associated with priming-enhanced root vigour. These findings highlight novel targets that could be leveraged in marker-assisted breeding to develop rapeseed cultivars with improved early root growth, which is crucial for robust seedling establishment.

**Supplementary Information:**

The online version contains supplementary material available at https://doi.org/10.1186/s12870-025-07540-4.

## Background

Seedling establishment represents a critical bottleneck in crop productivity, particularly under challenging environmental conditions such as erratic rainfall, poor soil fertility, and physical soil constraints. In *Brassica napus* L. (oilseed rape), insufficient early root vigour undermines crop establishment, impairs nutrient uptake, and ultimately limits yield, issues exacerbated by soil compaction and nutrient deficiency [1, 2]. Recent studies have highlighted the genetic and physiological mechanisms underpinning root system architecture (RSA) plasticity in *B. napus*, which plays a pivotal role in stress adaptation. For instance, Duan et al. in 2023 demonstrated that cultivars with large and deeper root mechanical traits in compacted soils exhibited improved water and nutrient foraging, leading to better seedling survival and establishment [3]. In addition, genetic mapping studies have also identified quantitative trait loci (QTLs) associated with root architectural traits such as primary root length, rate of primary root growth, lateral root length, and lateral root density, offering potential targets for breeding programmes [4]. Climate-adaptive root traits are also increasingly prioritised in breeding programmes, with evidence suggesting that root plasticity can buffer yield losses under fluctuating moisture regimes [5]. Furthermore, studies have shown that deep-rooted crops like *B. napus* enhance drought resilience by effectively exploring deeper soil layers, where root growth angle and root length are critical for accessing water and nutrients, contributing to more stable yields under water stress [6]. Thus, improving early root development emerges as a key strategy to enhance seedling establishment necessary for stress resilience, optimise resource acquisition, and promote sustainable yield gains, especially in the face of mounting climate variability and the need to reduce agrochemical inputs [7, 8].

Seed osmopriming, a pre-sowing technique that involves the controlled hydration of seeds in osmotic solutions, activates metabolic programs prior to germination without allowing radicle protrusion, has emerged as a promising technique to enhance germination uniformity and strengthen early root vigour [9]. It boosts physiological preparedness, enabling improved tolerance and recovery from abiotic stresses such as drought and suboptimal thermal conditions. This pre-sowing treatment also optimises metabolic efficiency, improving resource utilisation and maximising yield potential under variable environmental conditions [10]. Seed osmopriming has been investigated using a variety of osmotic agents, such as KCl, KNO₃, mannitol, CaCl₂, K₃PO₄, sorbitol and polyethylene glycol (PEG) [11, 12]. Among these, PEG has become one of the most widely adopted in plant physiological research [13]. Polyethylene glycol (PEG) is widely used in seed priming due to its chemical inertness, non-toxicity to seed embryos, and protein-stabilising properties, all of which enhance the reproducibility and efficacy of priming protocols. Furthermore, its high molecular weight prevents penetration into seed tissues, enabling the establishment of a controlled osmotic environment that regulates water uptake without direct interaction with cellular components [13–15]. These combined attributes make PEG a preferred choice for enhancing seed performance under a wide range of experimental and environmental conditions. Thus, by partially hydrating seeds in PEG solutions, pre-germinative metabolic processes, including cell cycle activation, reserve mobilisation, and hormonal signaling are initiated without radicle protrusion [16]. This initial metabolic induction primes seeds for rapid root growth upon sowing, thereby enhancing seedling establishment under stress conditions [17]. Osmopriming with PEG-6000 has been shown to enhance primary root length and seedling vigour in several crops under field or field-simulated conditions [18]. PEG-induced osmotic stress also modulates phytohormone signaling pathways, particularly those involving abscisic acid (ABA) and gibberellins (GA), which are critical for regulating germination [19]. In addition, osmopriming enhances the antioxidant defence system by stimulating reactive oxygen species (ROS) scavenging enzymes such as superoxide dismutase (SOD), catalase (CAT), and peroxidase (POD), thereby mitigating oxidative damage during early seedling development [20, 21]. The resulting improvement in ROS homeostasis supports cellular integrity and accelerates radicle elongation under stress. Recent studies have also suggested that osmopriming may influence the expression of genes related to stress perception, hormonal balance, and energy metabolism, indicating a complex and coordinated molecular response [9, 22–24].

As one of the world’s most widely cultivated oilseed crops, *Brassica napus* L., an allotetraploid species (AACC, 2n = 4X = 38), is a vital source of vegetable oil, biofuels, and animal feed [25]. It exists in three major morphotypes depending on vernalisation requirements: spring-type, semi-winter-type, and winter-type, each suited to different agro-climatic regions. Spring-type cultivars are primarily grown in Canada and northern Europe, winter-types are predominant in temperate regions such as western and central Europe, and semi-winter-types are extensively cultivated in China and parts of South Asia [25, 26]. However, poor seedling establishment remains a significant constraint, leading to yield losses due to uneven stands and poorly developed root systems [2]. While conventional breeding has focused on enhancing yield, flowering time, and oil content, early root vigour has received comparatively little attention, partly due to difficulties in phenotyping and the complex nature of genotype-by-environment interactions [27]. Genome-wide association studies (GWAS) offer a powerful tool to dissect the genetic architecture of root traits, as demonstrated in rice [28] and soybean [29]; however, the genetic mechanisms underlying phenotypic variation in osmopriming response remain poorly characterised in *B. napus*, limiting the effective integration of priming-responsive traits into elite cultivars. Identifying loci associated with priming responsiveness in root vigour traits could support marker-assisted selection for genotypes that benefit most from priming treatments, reducing the need for post-sowing interventions.

In this study, we employed genome-wide association (GWAS) to identify genetic loci associated with PEG-mediated osmopriming responses in spring-type *Brassica napus*. Root vigour traits evaluated included radicle emergence (RE, %), primary root length (RL, mm), root growth rate (RGR, mm/h), and vigour index (VI). To analyse marker–trait associations, we implemented the Bayesian-information and linkage-disequilibrium iteratively nested keyway (BLINK) model, a fixed-effects approach selected for its superior power and efficiency. BLINK integrates linkage disequilibrium-based marker clumping with dynamic covariate selection using the Bayesian Information Criterion (BIC), thereby improving correction for population stratification and reducing false positives compared with the Fixed and random model Circulating Probability Unification (FarmCPU) model [30]. Furthermore, its computational efficiency is advantageous for large-scale genomic analyses as it eliminates the need for kinship matrix inversion. To our knowledge, no prior GWAS has investigated osmopriming responses in *B. napus* or any other crop, underscoring the novelty of this work. We hypothesise that osmopriming induces measurable improvements in root vigour traits, with the extent of this response varying among genotypes, and predict that GWAS will uncover specific associated loci. Ultimately, these findings provide foundational insights into the genetic regulation of priming responses, supporting marker-assisted breeding for enhanced and synchronised root germination.

## Materials and methods

### Plant material, osmopriming treatment and growth conditions

An association panel comprising 228 genetically diverse spring-type *B. napus* inbred accessions was employed in this study. These accessions, which originated from 16 different geographical regions were sourced from the gene bank materials of Leibniz-Institut für Pflanzengenetik und Kulturpflanzenforschung, IPK (Gatersleben, Germany) and the breeding materials of Deutsche Saatveredelung AG, DSV (Lippstadt, Germany) (Supplementary1, Table S1). Seeds of all inbred accessions underwent two generations of controlled self-pollination. The first generation was conducted in greenhouse conditions during the 2021/2022 season, where two seeds per accession were sown and self-pollinated individually. From one of the resulting self-pollinated plants per accession, eighteen seeds were sown in the field during the 2022/2023 growing season at the Deutsche Saatveredelung AG (DSV) breeding station in Asendorf, Germany, where they were self-pollinated again and harvested to produce the final seed stock.

For the osmopriming treatment, clean seeds were soaked in a PEG-6000 solution (osmotic potential − 0.7 MPa, equivalent to 24% w/w at 25 °C) at a 1:2 seed-to-solution ratio for 24 h in darkness. The soaking was conducted in sealed plastic containers placed in a rotating drum while maintaining constant rotation to ensure proper mixing of the PEG-6000 solution with the seeds. This priming protocol was consistently employed in all subsequent analyses. Following priming, the seeds were transferred to a sieve and carefully washed several times for about 20 s with clean running tap water to remove the osmotic agent. After washing, the seeds were dried at ~ 28 °C for 24 h until they returned to their original weight, and subsequently either used directly for germination assay or stored under cold, low-humidity conditions for short-term preservation.

For the germination assay, seeds from each accession were placed in glass frame boxes. The boxes were lined with a layer of non-absorbent white foam, topped with two layers of filter paper. A blue filter at the top enhanced contrast, allowing for easier observation of root development. Before placing the seeds, the filter papers were thoroughly pre-soaked in distilled water to ensure consistent moisture levels throughout the germination process. This step helped create a uniform and optimal environment for seed hydration and germination. To ensure a fair comparison and minimise phenotyping bias, unprimed and primed seeds were placed side by side within the same glass frame, and different accessions were arranged together in a single frame. Each glass frame could hold four filter papers and seeds from six different accessions, with each filter paper containing three rows of seeds. Each row consisted of seeds from a single accession, ensuring an organised layout for consistent evaluation. The germination glass frames were securely closed and placed vertically in a dark chamber at a constant temperature of 20 °C, where the seeds were allowed to germinate for 72 h. All experiments were conducted at the Department of Seed Technology, Deutsche Saatveredelung AG Breeding Station, Asendorf, Germany.

### Phenotypic trait measurements

Root trait measurements in germinating seedlings were assessed using the CropScore large-scale phenotyping system (https://www.cropscore.com/home.html). Seedlings were imaged hourly, and the CropScore software automatically quantified root traits. Each accession was evaluated using 17 seedlings in each of two independent experimental runs conducted under highly controlled conditions. The mean values from each run were then recorded for analysis. A radicle was considered emerged when it reached 5 mm in length, as detected by the CropScore software. The assessed traits included radicle emergence (RE, %), root length (RL, mm), root growth rate (RGR, mm/h), and vigour index (VI), which was calculated using the formula: VI = RE (%) × RL (mm) [31]. RE, RL, and RGR were automatically tracked in real-time, with data generated and downloaded 72 h after sowing for further analysis upon completion of the experiment. The response to osmopriming treatment for each trait was evaluated using the relative response metric, which quantifies the genotypic performance following seed treatment compared to unprimed controls. The RR metric may be sensitive to very low baseline control values, which can likely inflate percentage changes.

The relative response, RR (%), was estimated using the following formula:

$$\mathit{Relative\ response,}\ RR(\%) = \frac{Trait_{primed} - Trait_{unprimed}}{Trait_{unprimed}} \times 100$$          

### DNA extraction, SNP genotyping and quality control

Genotypic data for 228 *Brassica napus* accessions were generated using the 19 K *Brassica* Infinium SNP array (TraitGenetics, SGS INSTITUT FRESENIUS GmbH, Gatersleben, Germany), which comprises 18,566 single-nucleotide polymorphisms (SNPs). This array was employed in strict accordance with the manufacturer’s protocols. Genomic DNA was isolated from young leaves of eight seedlings per accession using an internally validated extraction protocol that utilised cetyltrimethylammonium bromide (CTAB) in combination with dichloromethane to optimise purity and yield. Subsequent genotyping was performed on the Illumina Infinium XT platform (Illumina Inc., San Diego, CA). It is noteworthy to state that a substantial proportion of the SNP markers incorporated in this array originates from the already established *Brassica* 60 K Illumina Infinium array [32], thereby ensuring comprehensive genomic coverage and facilitating robust comparative analyses. Following data acquisition, rigorous quality control measures were implemented. SNPs with a minor allele frequency (MAF) < 5% or a call rate below 90% were excluded from the dataset. This filtering process resulted in the retention of 15,311 high-quality SNP markers for downstream genome-wide association study (GWAS) analyses (Supplementary1, Table S2). Of these markers, 8,162 were attributed to the A-subgenome, while 7,149 corresponded to the C-subgenome, enabling detailed subgenomic investigations (Supplementary1, Table S4; Supplementary2, Fig. S1).

### Population structure, principal component analysis & linkage disequilibrium (LD)

Population structure was evaluated using Bayesian clustering of multilocus allele frequencies, implemented in STRUCTURE software, v.2.3.4 [33]. The analysis employed the filtered SNP dataset, evaluating number of clusters (*K*) from 1 to 10, with five independent replicate runs performed for each *K*. Each run consisted of a 10,000-iteration burn-in period followed by 20,000 Markov chain Monte Carlo (MCMC) simulations. The optimal number of clusters (*K*) was identified using STRUCTURE HARVESTER by selecting the value associated with the highest *ΔK* [34, 35]. Individuals with ancestry coefficients > 50% were assigned to distinct subpopulations. Principal component analysis (PCA) was performed using the Trait Analysis by aSSociation, Evolution and Linkage (TASSEL v5.0) software [36], and the resulting components were visualised with scatterplots generated in R via the *ggplot2* package. These principal components were incorporated as covariates in the genome-wide association study (GWAS) to adjust for population stratification. Kinship matrices were also computed in parallel using VanRaden’s method with the *GAPIT3* package in R [37]. Linkage disequilibrium (LD) between SNP pairs was assessed as *r²* using also TASSEL v5.0. LD block sizes were determined for both A-subgenome and C-subgenome and the whole genome, with the *r²* threshold (*r*^*2*^ = 0.2) set to correspond to the observed LD decay. LD patterns around the GWAS peaks were generated and visualised using the *LDheatmap* package in R.

### Genome-wide association analysis (GWAS), identification of candidate genes and haplotype analysis

Genome-wide association studies (GWAS) were performed using the *GAPIT3* package in R, implementing the BLINK (Bayesian-information and Linkage-disequilibrium Iteratively Nested Keyway) model to leverage its superior statistical power and reduced false positive rate [30]. Population stratification was accounted for by incorporating the first three principal components (PCs) from principal component analysis (PCA) as fixed covariates. Additionally, a kinship matrix was estimated using the VanRaden method to provide an unbiased measure of genetic relatedness among individuals. Genome-wide marker-trait associations were identified using a Bonferroni-adjusted significance threshold (*p* ≤ 3.29 × 10⁻⁶, equivalent to − log₁₀(*p*) ≥ 5.48) and a false discovery rate (FDR) < 0.05. Additionally, SNPs with suggestive significance (*p* ≤ 1 × 10⁻^4^, equivalent to − log₁₀(*p*) ≥ 4) were evaluated for their potential biological relevance to osmopriming response, as they may represent secondary signals or contribute to polygenic adaptation. Candidate genes associated with significant SNPs were determined via a BLAST mapping strategy. The genomic regions surrounding these SNPs were aligned against the *Brassica napus* ‘Darmor-bzh’ reference genome using the BnIR JBrowser (https://yanglab.hzau.edu.cn/BnIR), and homologous genes in *Arabidopsis thaliana* were identified through BLAST analysis [25, 38]. Candidate gene search was defined using a 400 kb window, spanning ± 200 kb from each lead SNP [39]. This window was chosen to capture genes potentially linked to the trait of interest while limiting the inclusion of distant, less relevant loci. Genes within this window were functionally annotated for gene ontology terms using ShinyGO v0.82 (https://bioinformatics.sdstate.edu/go/), based on *Arabidopsis thaliana* gene IDs and the Araport11 genome database [40]. Haplotype analysis was conducted using phased genotypic data derived from GWAS-identified SNPs. Haplotype blocks were defined using the confidence interval method described by Gabriel et al. [41], as implemented in Haploview software (v4.2). To ensure robust comparisons, haplotypes represented by fewer than five individuals were excluded from downstream analysis.

### Statistical analysis

All statistical analyses were conducted using R (version 4.4.1). Descriptive statistics were generated via built-in functions. For the comparison of phenotypic data, we applied either the Student’s t-test or Wilcoxon’s test, based on whether the data met the assumptions of normality and homogeneity of variances or not. Pearson correlation coefficients among traits were determined with the *pairs.panel* function from the *psych* package. Principal component analysis was performed using the *prcomp* function from the stats package. The *lme4* package in R was utilised in performing ANOVA and estimating heritability components.

For each trait, a linear mixed-effects model was fitted to evaluate the effects of treatment and genotype while accounting for experimental replicates. The model was specified as:

$$Y_{ijk}=\mu+T_i+G_j+{\left(T\times G\right)}_{ij}+R_k+\varepsilon_{ijk}\\$$  

where Y_ijk_ ​is the observed value of the trait for the j^th^ accession under the i^th^ treatment in the k^th^ replicate; µ is the overall mean of the trait; T_i_​ is the effect of the i^th^ treatment; G_j_ is the effect of the j^th^ accession (genotype); (T×G)_ij_​ is the effect of the interaction between treatment and accession; R_k_​ is the effect of the k^th^ replicate, assumed to be normally distributed with mean 0 and variance σ^2^​_R_; ε_ijk​_ is the residual error term, assumed to be normally distributed with mean 0 and variance σ^2^.

Broad-sense heritability (*H²*) was estimated with the formula:

$$H^2=\frac{\sigma^2G}{(\sigma^2G+\sigma^2E/r)}$$  

where *σ²*G is the genetic variance; *σ²*E is the error variance; r is the replication number.

While the phenotypic variance explained, PVE (%) values of SNPs that exceeded the genome-wide significant threshold were estimated by the BLINK model, the naïve PVE (%) of SNPs that only crossed the suggestive threshold were calculated using the formula:

$$PVE(\%) = \frac{2p(1-p)\beta^2}{Var(y)}\,\times\,100$$  

Where *p* is the minor allele frequency of the SNP; *β* is the effect size of the SNP (from BLINK output); Var(*y*) is the total variance of the phenotype [42]

For the statistical evaluation of haplotype effects, the Kruskal-Wallis test followed by Dunn’s post hoc test was applied since the assumptions of normality of residuals and homogeneity of variance were not met.

## Results

### Phenotypic variation in root vigour traits and relative response to osmopriming

**Fig. 1:**
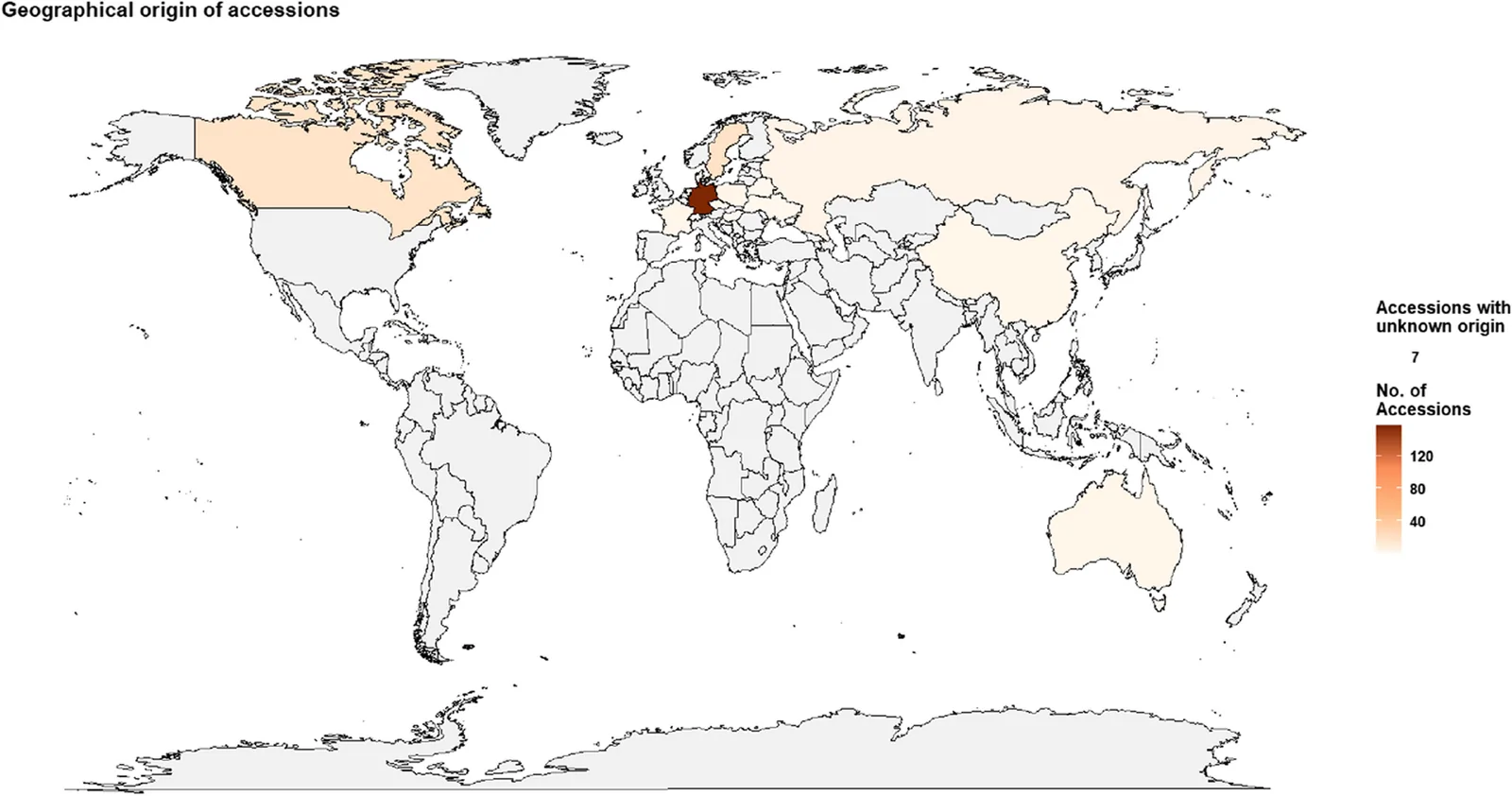
Geographic distribution of the accessions analysed in this study. The provided scale indicates the number of accessions originating from each country

**Fig. 2:**
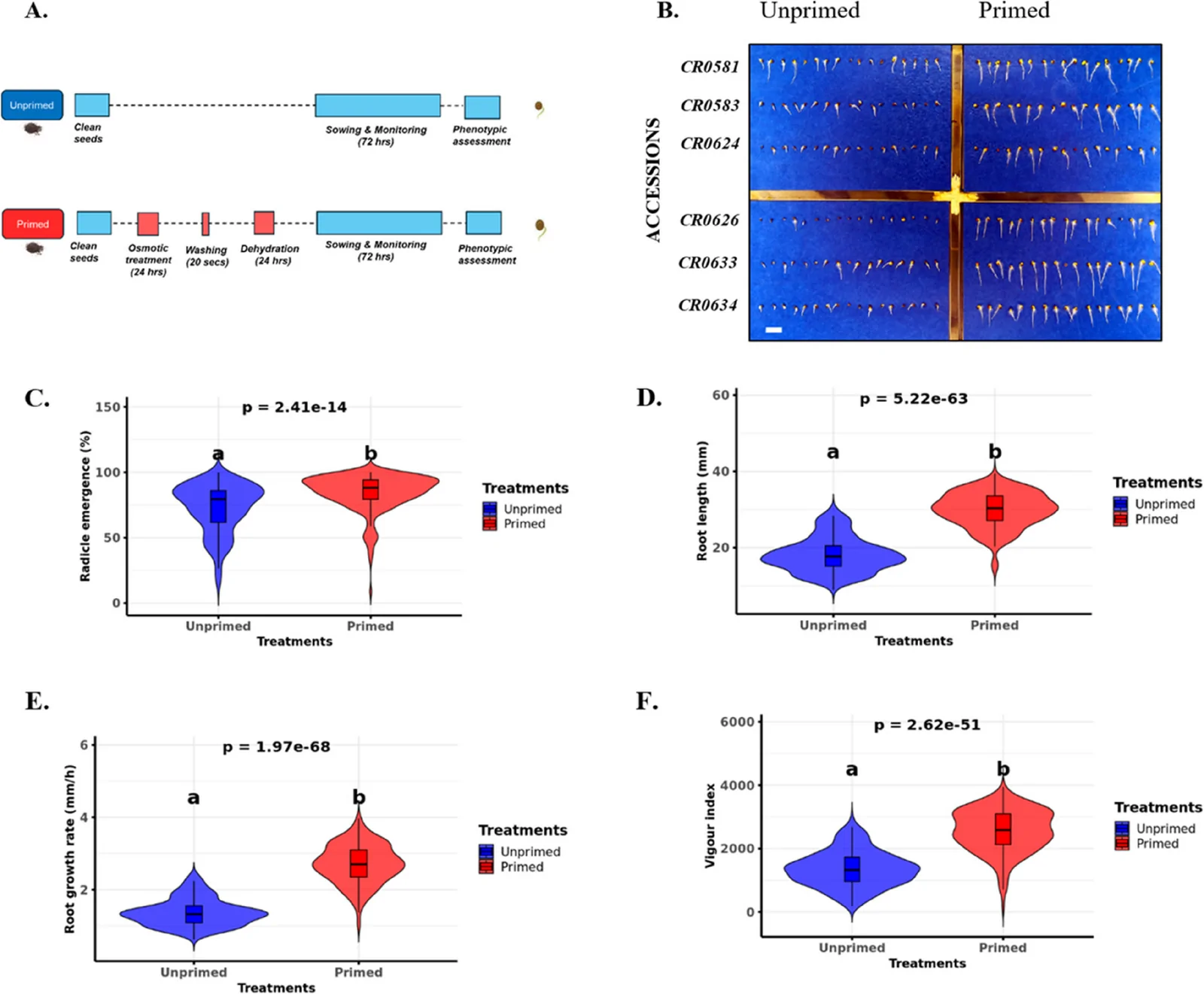
Effects of seed osmopriming on radicle development and root vigour across 228 accessions. **A** Schematic diagram of unprimed and primed treatments; **B** representative radicle images from six accessions under both treatments after 48 h of sowing (scale bar = 25 mm); **C** radicle emergence (RE); **D** root length (RL); **E** root growth rate (RGR); and **F** vigour index (VI), each shown as violin plots comparing unprimed and primed seeds across all accessions. Distinct letters displayed above the violin plots represent statistically significant differences (*p <* 0.05) between groups, determined by pairwise comparisons

To investigate phenotypic variation in root vigour traits and their response to osmopriming, we evaluated a genetically diverse association panel consisting of 228 spring-type *Brassica napus* inbred accessions collected from a broad range of geographical origins (Fig. 1). The seeds of all accessions were treated with PEG-6000 solution, and the relative response of each accession was quantified in comparison to its respective unprimed control (Fig. 2a). Phenotypic assessments included measurements of radicle emergence (RE), root length (RL), root growth rate (RGR), and vigour index (VI) under both unprimed and primed conditions to characterize the extent of variation in seedling performance across the panel. Osmopriming significantly enhanced all measured traits, supporting its effectiveness in improving early seedling establishment (Fig. 2b; Table 1). Mean RE increased from 72.65 ± 18.71% (unprimed) to 84.38 ± 14.15% (primed), representing a 16.1% absolute mean increase (*p <* 0.0001). RL showed a marked improvement of 64.6%, increasing from 18.15 ± 4.54 mm to 29.87 ± 4.61 mm (*p <* 0.0001). RGR nearly doubled from 1.37 ± 0.38 to 2.71 ± 0.54 mm/h (*p <* 0.0001), while VI increased from 1355.06 ± 561.13 to 2556.02 ± 671.26 units (*p <* 0.0001) (Fig. 2c–f; Table 1). These improvements collectively demonstrate the positive impact of osmopriming on root system development. In addition to enhancing mean trait values, priming reduced phenotypic variability within the population, as shown by a decrease in the coefficient of variation (CV) for all traits: RE (from 25.8% to 16.8%), RL (from 25.0% to 15.5%), RGR (from 27.5% to 19.9%), and VI (from 41.4% to 26.3%) (Table 1; Supplementary1, Table S3). Heritability (*H²*) estimates were generally moderate to high but showed a varied response upon priming. While *H²* for VI remained seemingly stable (0.72 unprimed vs. 0.69 primed), the estimates for RE (0.41 unprimed vs. 0.28 primed), RL (0.69 unprimed vs. 0.55 primed), and RGR (0.69 unprimed vs. 0.30 primed) decreased. (Supplementary1, Table S3). Crucially, the relative response (RR) metric further revealed substantial phenotypic variation in osmopriming response (Table 1). Mean RR values were + 26.6% for RE, + 72.1% for RL, + 113.7% for RGR, and + 124.2% for VI. However, we observed wide RR ranges across all genotypes for the measured root traits (e.g., RR-VI from − 90.1% to + 965.1%). The large standard deviations and coefficients of variation in RR values (e.g., 99.69% CV for RR-VI) underscore the diversity of osmopriming responsiveness within the population (Table 1; Supplementary1, Table S3). Together, these results demonstrate that PEG-mediated osmopriming not only improves root vigour traits but also reveals substantial variation in relative response, highlighting the potential to genetically dissect and harness priming responsiveness for breeding more resilient seedling phenotypes.

**Table 1:** Descriptive statistics values for root vigour traits of unprimed and primed seedlings and their relative responses to osmopriming

Traits	Treatments	Mean ± SD	Min.	Max.	CV (%)	Range	Kurtosis	Skewness
RE (%)	Unprimed	72.65 ± 18.71	14.71	100.00	25.76	85.30	0.26	−0.93
	Primed	84.38 ± 14.15	8.82	100.00	16.77	91.18	4.85	−1.88
RL (mm)	Unprimed	18.15 ± 4.54	8.96	30.39	25.03	21.43	−0.18	0.55
	Primed	29.87 ± 4.61	14.20	39.44	15.45	25.24	0.36	−0.61
RGR (mm/h)	Unprimed	1.37 ± 0.38	0.62	2.43	27.45	1.80	0.02	0.63
	Primed	2.71 ± 0.54	1.05	3.97	19.90	2.92	−0.01	−0.27
VI	Unprimed	1355.06 ± 561.13	183.22	2949.17	41.41	2765.94	−0.18	−0.12
	Primed	2556.02 ± 671.26	141.79	3944.00	26.26	3802.21	0.33	−0.61
RR-RE (%)	-	26.56 ± 51.42	−89.66	380.04	193.64	469.70	14.94	3.09
RR-RL (%)	-	72.08 ± 39.58	−21.15	181.86	54.92	203.01	−0.32	0.33
RR-RGR (%)	-	113.67 ± 53.57	−7.21	287.40	47.13	294.62	−0.11	0.36
RR-VI (%)	-	124.23 ± 123.84	−90.14	965.07	99.69	1055.21	12.33	2.71

*Min *Minimum, *Max* Maximum, *SD* Standard deviation, *CV* Coefficient of variance

### Pearson’s correlation and principal component analysis of relative response to osmopriming

**Fig. 3:**
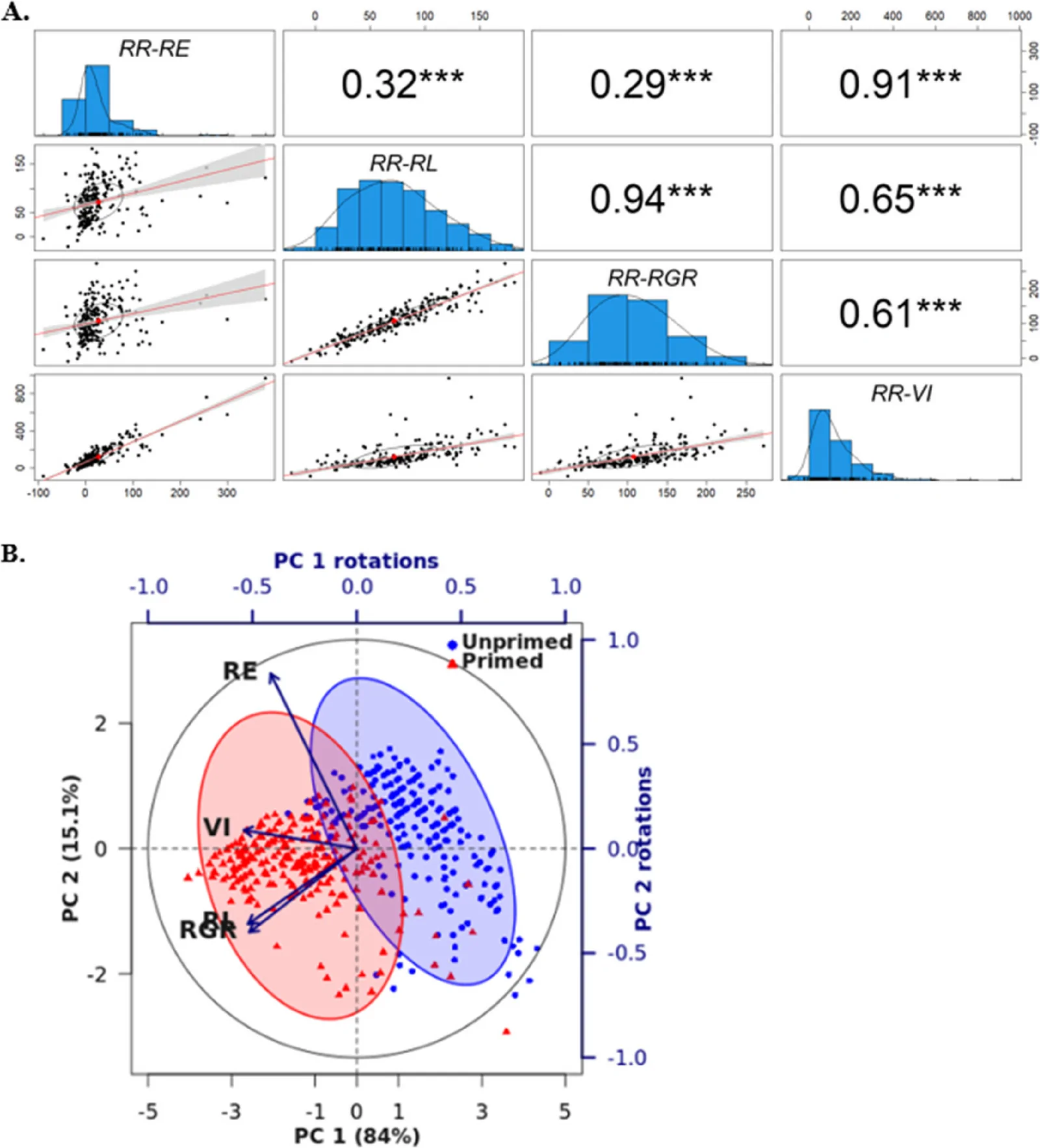
Phenotypic correlation and principal component analysis of relative response to osmopriming. **A** Correlation analysis and frequency distributions of osmopriming response among four root vigour traits. The numbers in the boxes indicate the correlation coefficients (R-values) between the traits. The histograms represent the trend of distribution of each trait **B** PCA biplot showing primed vs. unprimed seedlings based on four root vigour traits. RR-RE = relative response for radicle emergence; RR-RL = relative response for root length; RR-RGR = relative response for root growth rate; RR-VI = relative response for vigour index. *** represents significance at the *p <* 0.001 level

To examine the relationships among root vigour traits in response to osmopriming, we performed Pearson’s correlation analysis on the relative responses of all four root vigour traits. Our analysis revealed highly significant associations (*p <* 0.001) among the accessed root vigour traits (Fig. 3a). The osmopriming response for root length (RR-RL) correlated strongly and positively with that for relative growth rate (RR-RGR) (*r* = 0.94, *p <* 0.001). Similarly, the response for root emergence (RR-RE) correlated strongly with the response for vigour index (RR-VI) (*r* = 0.91, *p <* 0.001). We found moderately positive correlations of RR-VI with both RR-RL (*r* = 0.65, *p <* 0.001) and RR-RGR (*r* = 0.61, *p <* 0.001). In contrast, the correlations of RR-RE with RR-RL (*r* = 0.32, *p <* 0.001) and RR-RGR (*r* = 0.29, *p <* 0.001) were weaker, though still significant. This correlation pattern suggests that while these traits are interrelated, the relationship between early root emergence and subsequent growth is less direct under these priming conditions.

Principal component analysis (PCA) of root vigour traits distinguished unprimed and primed seedlings, with the first two principal components accounting for 99.1% of the total variation (PC1 = 84.0%, PC2 = 15.1%) (Fig. 3b). The clear clustering of treatments along PC1 indicates that osmopriming was a major source of variation. All measured traits loaded positively on PC1 for the primed group. The vectors for RL and RGR showed the strongest loadings on PC1, indicating they were the traits most associated with the separation between treatments.

### Population structure and linkage disequilibrium of the association panel

**Fig. 4:**
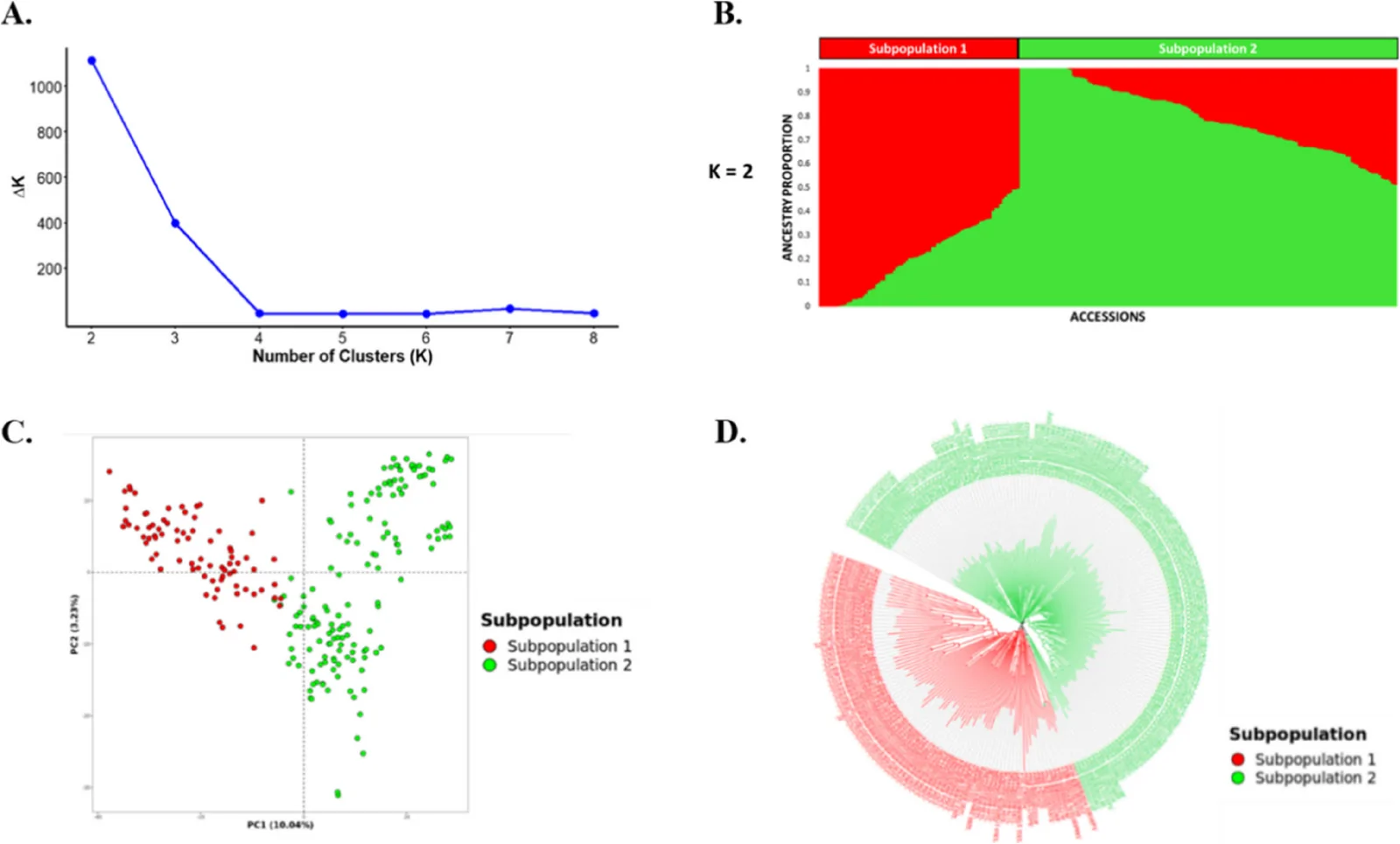
Population structure analysis of the association panel **A** Delta *K* plot showing the number of subpopulations present in the association panel **B** Subpopulations present in the association panel at *K* = 2. **C** 2D PCA plot elaborating subpopulations as identified by the PCA analysis **D** NJ tree of accessions in the association panel

**Fig. 5:**
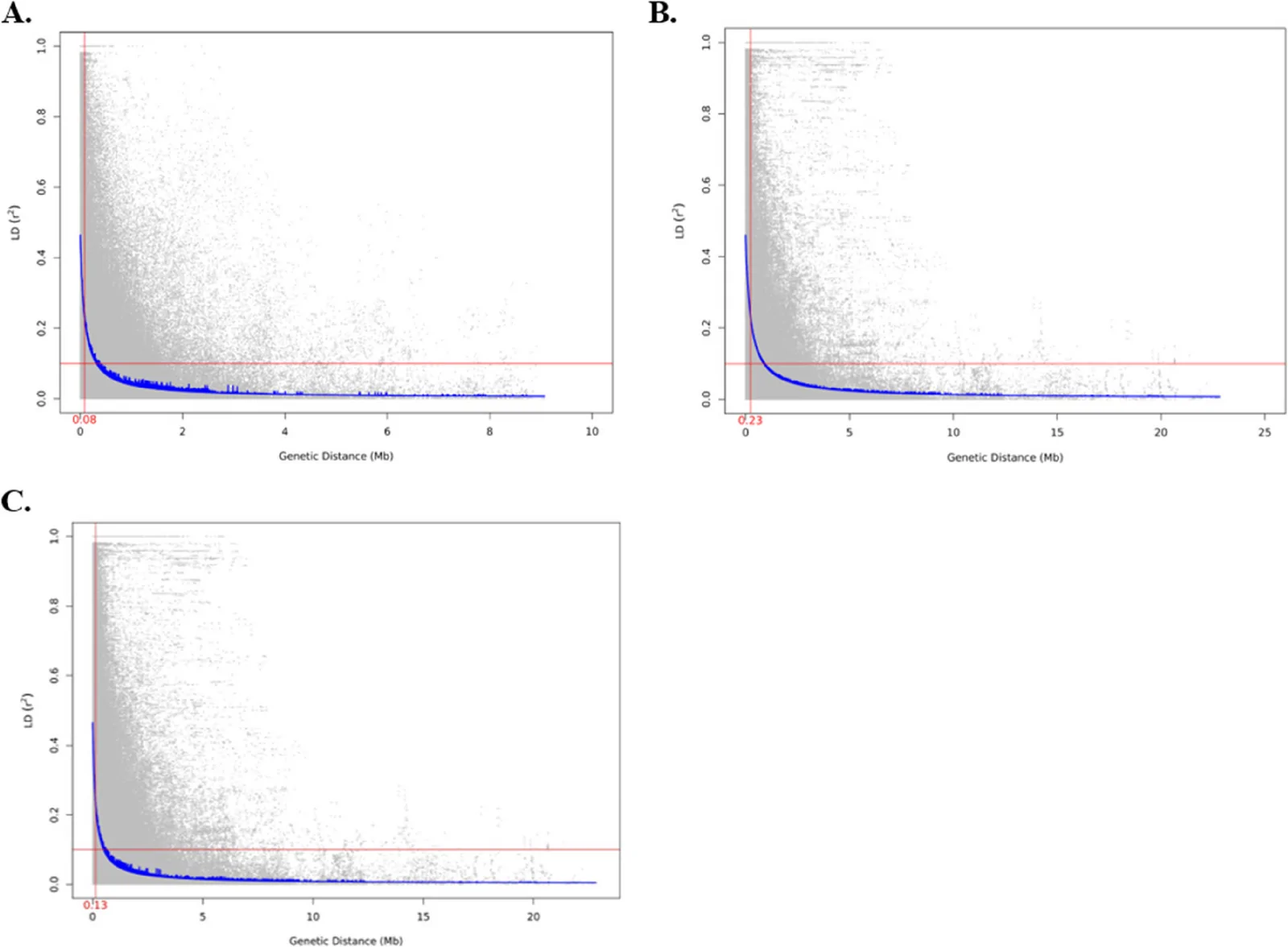
Scatter plot of linkage disequilibrium (LD) decay, showing mean ***r²*** values plotted against physical distances between markers (Mb) **A **A-subgenome **B** C-subgenome **C** Whole genome. The blue curve illustrates the LD decay pattern modelled using a smoothing spline regression. The vertical red line shows the position where the decay curve meets the half-decay threshold at *r*^*2*^ = 0.2. The horizontal red line marks the genome-wide half-decay *r*^*2*^ value

We evaluated the number of clusters (*K*) against the corresponding *ΔK* values to determine the optimal number of genetic subpopulations. While both *K* = 2 and *K* = 3 showed notable *ΔK* values, the highest peak was observed at *K* = 2, indicating that two subpopulations best represent the underlying genetic structure (Fig. 4a). STRUCTURE analysis classified the 228 *Brassica napus* accessions into two distinct groups: Subpopulation 1 and Subpopulation 2 based on the optimal clustering at *K* = 2 (Fig. 4b), however, results for *K* = 3 were also visualized to explore potential substructure (Supplementary2, Fig. S2). Subpopulation 1 was predominantly composed of modern breeding lines from the DSV program with 79 accessions, including 76 from DSV breeding lines and only 3 accessions from the IPK gene bank. In contrast, subpopulation 2 was a more admixed group, consisting 149 accessions, of which 84 were sourced from the IPK gene bank and 65 from DSV breeding lines (Supplementary1, Table S5). Principal component analysis (PCA) based on genome-wide SNP markers revealed that the first and second components explained 10.04% and 3.23% of the total genetic variation, respectively, effectively capturing the genetic structure among the accessions (Fig. 4c; Supplementary2, Fig. S3). Additionally, nearest neighbour clustering using imputed SNP markers provided a clear separation between subpopulation 1 and subpopulation 2, reinforcing the population structure revealed by the previous analyses (Fig. 4d). Kinship-based hierarchical clustering was also visualised as a heatmap of the pairwise relatedness matrix (Supplementary2, Fig. S4). To assess patterns of linkage disequilibrium (LD), the squared correlation coefficient (*r²*) was plotted against physical distance (Mb). Notably, the LD patterns of the A- and C-subgenomes showed clear distinctions across all chromosomes. (Supplementary1, Table S4). Furthermore, the A-subgenome (8,162 SNPs) displayed a rapid LD decay, with a half-life of 0.08 Mb (~ 80 kb), indicating relatively weak haplotype persistence (Fig. 5a). In contrast, the C-subgenome (7,149 SNPs) exhibited stronger LD, with a half-life of 0.23 Mb (~ 230 kb), nearly three times slower decay than the A-subgenome (Fig. 5b). Genome-wide LD decay had an intermediate half-life of 0.13 Mb (~ 130 kb), reflecting the composite structure of *B. napus* subgenomes (Fig. 5c).

### Genome-wide marker-trait associations for response to osmopriming

**Fig. 6:**
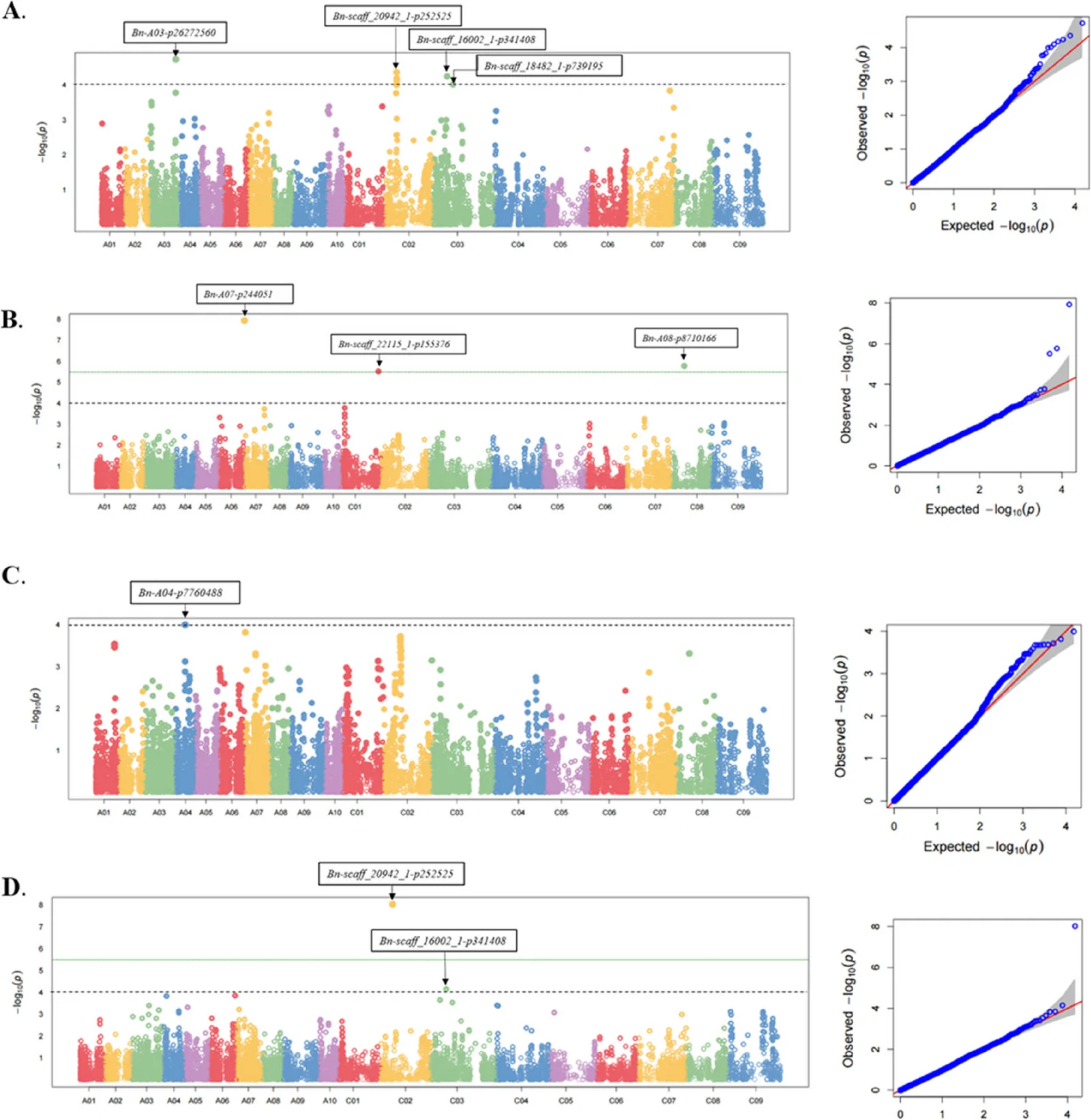
Genome-wide association studies for relative response to osmopriming. Manhattan and quantile–quantile plots for (A) RR-RE (B) RR-RL (C) RR-RGR (D) RR-VI. The green horizontal line represents the Bonferroni-adjusted significance threshold (− log₁₀*(p)* = 5.48). The black dashed line represents the suggestive significance threshold (− log₁₀*(p)* = 4)

We conducted genome-wide association studies (GWAS) to identify genomic regions significantly associated with the osmopriming response, using relative response metrics derived from four root vigour traits of unprimed and osmoprimed seedlings (i.e., RR-RE, RR-RL, RR-RGR & RR-VI). Each metric was analysed independently to capture trait-specific genetic associations linked to osmopriming. The BLINK fixed model was employed for its enhanced power in detecting associations while controlling for population structure [30]. Our analysis detected eleven SNPs associated with the osmopriming response across seven chromosomes (A03, A04, A07, C01, C02, C03 and C08) (Fig. 6). Four of these SNPs exceeded the genome-wide significance threshold (–log₁₀ (*p)* ≥ 5.48; FDR < 0.05), while seven additional SNPs reached suggestive significance (–log₁₀ *(p)* ≥ 4). These associated SNPs were located in intronic, exonic, intergenic, and untranslated regions (Table 2). For RR-RE, we identified seven SNPs on chromosomes A03 (1), C02 (4), and C03 (2) (Fig. 6a). To avoid redundancy from linkage disequilibrium, we retained only the lead SNP from the cluster of four closely located SNPs on chromosome C02 for downstream analysis. Three SNPs associated with RR-RL were localised to chromosomes A07, C01, and C08, respectively (Fig. 6b). A single significant SNP for RR-RGR was found on chromosome A04 (Fig. 6c), while two SNPs associated with RR-VI were detected on chromosomes C02 and C03 (Fig. 6d). We found that the SNPs *Bn-scaff_20942_1-p252525* (chromosome C02) and *Bn-scaff_16002_1-p341408* (chromosome C03) were significantly associated with both RR-RE and RR-VI. This co-localization of associations may reflect the contribution of RR-RE to the overall RR-VI measure, as RE constitutes a component of VI (Fig. 6; Table 2). The percentage of phenotypic variance explained (PVE, %) by the associated SNPs ranged from 2.49% (*Bn-scaff_16002_1-p341408*, associated with RR-VI) to 25.66% (*Bn-scaff_22115_1-p155376*, associated with RR-RL) (Table 2). A total of 342 candidate genes located within the LD intervals of the significant SNPs were successfully annotated, thereby implicating these regions in the genetic architecture of osmopriming response (Supplementary1, Table S6). Among these, we prioritised 23 genes as high-confidence candidates based on their physical linkage to lead SNPs within conserved LD blocks and functional relevance to osmotic stress adaptation, redox homeostasis, root development, and hormone-mediated drought responses. These candidates included homologs of *Arabidopsis* genes with established roles in stress-responsive root plasticity (Table 3).

**Table 2:** Marker trait associations detected for response to osmopriming in four root related traits using the BLINK GWAS model

SNP_id	Traits	Alleles	Chromosomes	Position (bp)	MAF	Genomic location	*P*-value	FDR	Effect size	PVE (%)
Bn-A03-p26272560	RR-RE	A/G	A03	24,660,653	0.37	BnaA03g47930D (Exon 12)	1.88E-05	0.22	−18.67	6.12
Bn-A04-p7760488	RR-RGR	T/C	A04	9,078,490	0.4	Intergenic	1.02E-04	0.38	15.01	4.14
Bn-A07-p244051^*^	RR-RL	T/C	A07	206,928	0.1	BnaA07g00200D (Intron 8)	1.17E-08	1.78E-04	−21.42	18.82
Bn-scaff_22115_1-p155376^*^	RR-RL	A/G	C01	35,743,833	0.1	BnaC01g36460D (Exon 4)	1.69E-06	1.55E-02	−17.94	25.66
Bn-scaff_20942_1-p252525^*^	RR-RE	A/G	C02	11,015,784	0.34	Intergenic	4.43E-05	0.22	17.94	5.46
	RR-VI						9.61E-09	1.46E-04	48.69	15.43
Bn-scaff_20942_1-p125124	RR-RE	A/G	C02	11,131,769	0.33	Intergenic	7.97E-05	0.22	17.27	5
Bn-scaff_20942_1-p121935	RR-RE	A/G	C02	11,139,601	0.33	Intergenic	1.02E-04	0.22	17.19	4.97
Bn-scaff_20942_1-p69491	RR-RE	A/G	C02	11,175,295	0.34	BnaC02g15550D (UTR)	6.61E-05	0.22	17.53	5.2
Bn-scaff_16002_1-p341408	RR-RE	A/G	C03	13,967,249	0.07	BnaC03g24850D (Exon 5)	5.77E-05	0.22	−27.74	4.02
	RR-VI						7.16E-05	0.54	52.56	2.49
Bn-scaff_18482_1-p739195	RR-RE	T/C	C03	19,626,762	0.08	BnaC03g31900D (Intron 1)	9.84E-05	0.22	−26.91	3.88
Bn-A08-p8710166^*^	RR-RL	T/C	C08	12,464,832	0.1	BnaC08g08380D (Intron 3)	3.08E-06	1.29E-02	19.03	12.21

*MAF * minor allele frequency, *bp* base pair, *PVE* phenotypic variance explained, *UTR * untranslated region, ^*^ = SNP markers exceeding genome-wide significance threshold and false discovery rate *(FDR) < 0.05.* SNPs *Bn-scaff_20942_1-p252525* and *Bn-scaff_16002_1-p341408* were detected in both RR-RE and RR-VI

**Table 3:** Putative candidate genes associated with osmopriming response in root vigour traits

SNP_id	Traits	Chr	Candidate genes	Arabidopsis homologue	Genomic position	Annotation	References
Bn-A03-p26272560	RR-RE	A03	BnaA03g47830D	AT4G26080	chrA03:24,563,270–24,566,288	ABA INSENSITIVE 1 (*ABI1*); Protein phosphatase 2 C	[54, 72]
	RR-RE	A03	BnaA03g47820D	AT4G26070	chrA03:24,560,638–24,563,048	MAP kinase/ERK kinase 1 (*MEK1*)	[72]
	RR-RE	A03	BnaA03g47930D	AT4G26300	chrA03:24,659,340–24,663,144	Embryo defective 1027 (*emb1027*); Arginyl-tRNA synthetase, class Ic	[56]
	RR-RE	A03	BnaA03g47860D	AT4G26150	chrA03:24,590,485–24,592,208	Cytokinin-responsive GATA factor 1 (*CGA1*)	[56]
	RR-RE	A03	BnaA03g47870D	AT4G26150	chrA03:24,596,130–24,597,812	Cytokinin-responsive GATA factor 1 (*CGA1*)	[56]
Bn-A04-p7760488	RR-RGR	A04	BnaA04g10190D	AT5G40330	chrA04:8,997,151–8,999,910	Myb domain protein 23 (*MYB23*)	
	RR-RGR	A04	BnaA04g10260D	AT5G40390	chrA04:9,043,551–9,047,080	Seed imbibition 1-like (*SIP1*)	[60]
	RR-RGR	A04	BnaA04g10280D	AT5G40440	chrA04:9,054,950–9,057,786	Mitogenactivated protein kinase kinase 3 (*MKK3*)	
Bn-A07-p244051	RR-RL	A07	BnaA07g00140D	AT1G49320	chrA07:136,262–140,175	unknown seed protein like 1 (*USPL1*); BURP	
	RR-RL	A07	BnaA07g00150D	AT2G20270	chrA07:172,207–173,454	Thioredoxin superfamily protein (*GRXS12*)	[70]
	RR-RL	A07	BnaA07g00240D	AT2G20340	chrA07:236,484–239,481	Pyridoxal phosphate (*PLP*) dependent transferases superfamily protein	
	RR-RL	A07	BnaA07g00200D	AT2G20320	chrA07:204,101–209,217	*DENN* (*AEX-3*) domain containing protein	[78]
Bn-scaff_22115_1-p155376	RR-RL	C01	BnaC01g36460D	AT3G14950	chrC01:35,742,394–35,745,260	Tetratricopetiderepeat thioredoxinlike 2 (*TTL2*)	
	RR-RL	C01	BnaC01g36330D	AT3G15210	chrC01:35,650,908–35,651,657	Ethylene responsive element binding factor 4 (*ERF4*)	[79]
	RR-RL	C01	BnaC01g36430D	AT3G15030	chrC01:35,696,597–35,697,693	TCP family transcription factor 4 (*TCP4*)	[76]
Bn-scaff_20942_1-p252525	RR-RE, RR-VI	C02	BnaC02g15510D	AT5G51810	chrC02:11,109,631–11,111,035	Gibberellin 20 oxidase 2 (*GA20ox2*)	[63]
	RR-RE, RR-VI	C02	BnaC02g15500D	AT5G51890	chrC02:11,056,194–11,057,360	Peroxidase superfamily protein (*PER66*)	[70]
	RR-RE, RR-VI	C02	BnaC02g15440D	AT5G51950	chrC02:11,001,007–11,003,773	Glucosemethanolcholine (GMC) oxidoreductase family protein (*MSG15.3*)	[46]
Bn-scaff_16002_1-p341408	RR-RE, RR-VI	C03	BnaC03g24850D	AT2G44510	chrC03:13,966,672–13,968,097	CDK inhibitor P21 binding protein	
Bn-scaff_18482_1-p739195	RR-RE	C03	BnaC03g31730D	AT4G01026	chrC03:19,523,341–19,524,503	Abscisic acid receptor (*PYR1*)	[53]
	RR-RE	C03	BnaC03g32130D	AT4G00430	chrC03:19,779,031–19,780,668	Plasma membrane intrinsic protein 1;4 (*PIP1;4*);	[58, 59]
Bn-A08-p8710166	RR-RL	C08	BnaC08g08390D	AT4G14130	chrC08:12,467,531–12,468,650	Xyloglucan endotransglucosylase/hydrolase 15 (*XTH15*)	[63, 71, 72]
	RR-RL	C08	BnaC08g08380D	-	chrC08:12,464,000–12,466,009	-	

### Identification of favourable haplotypes associated with osmopriming response

**Fig. 7:**
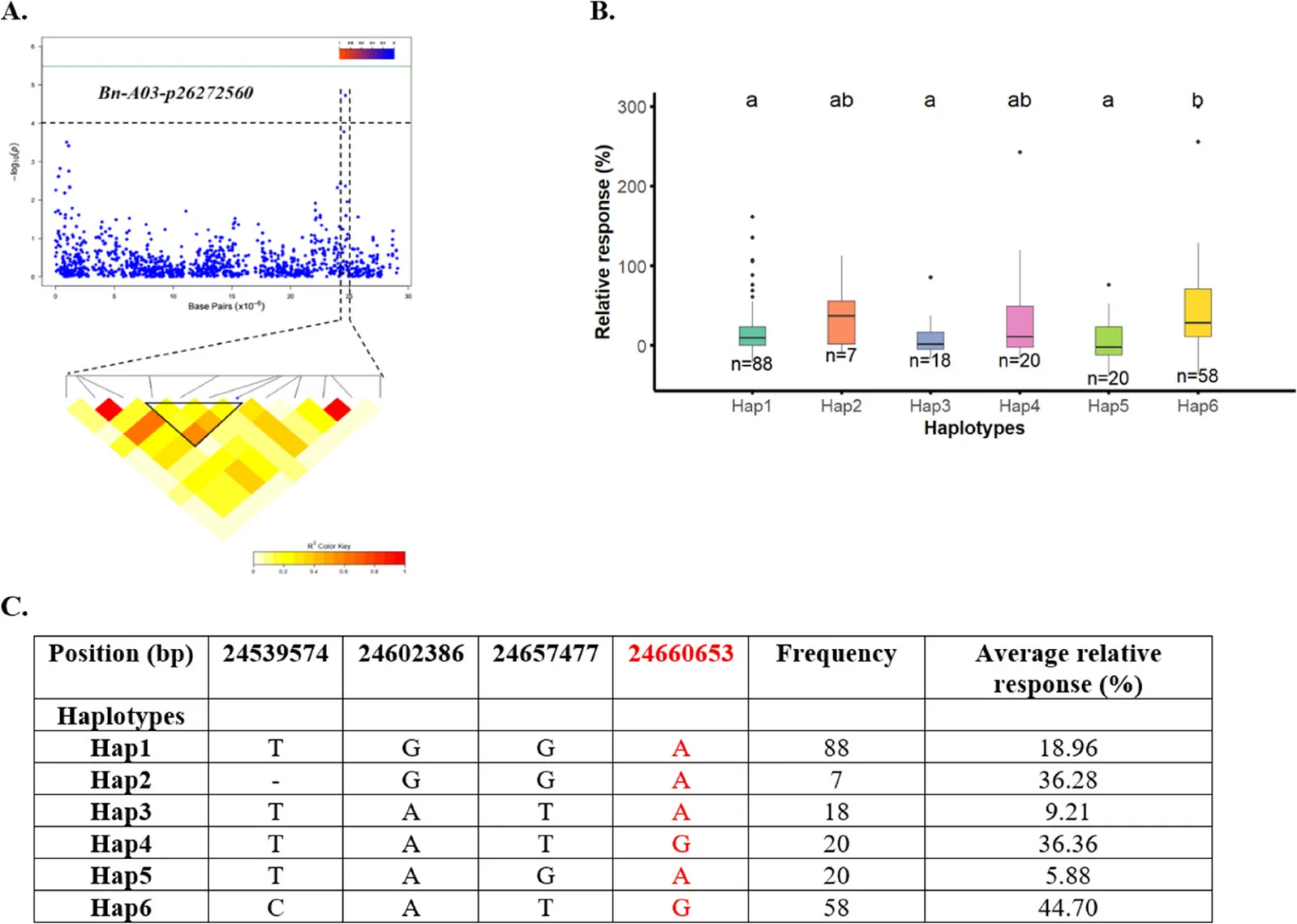
GWAS results and haplotype analysis for ***SNP Bn-A03-p26272560*** associated with RR-RE. **A** A Manhattan plot illustrating RR-RE is shown. The green and black horizontal lines mark the Bonferroni & suggestive significance threshold, respectively. Linkage disequilibrium (LD) among SNPs on chromosome A03 is displayed below, based on pairwise *r²* values, which are color-coded according to the provided scale. **B** Phenotypic differences in osmopriming response (RR-RE) between the identified haplotypes **C** Haplotype analysis of *SNP Bn-A03-p26272560* (in red text) and three other linked SNPs. Six haplotypes (Hap1, Hap2, Hap3, Hap4, Hap5 and Hap6) were identified in 211 accessions

**Fig. 8:**
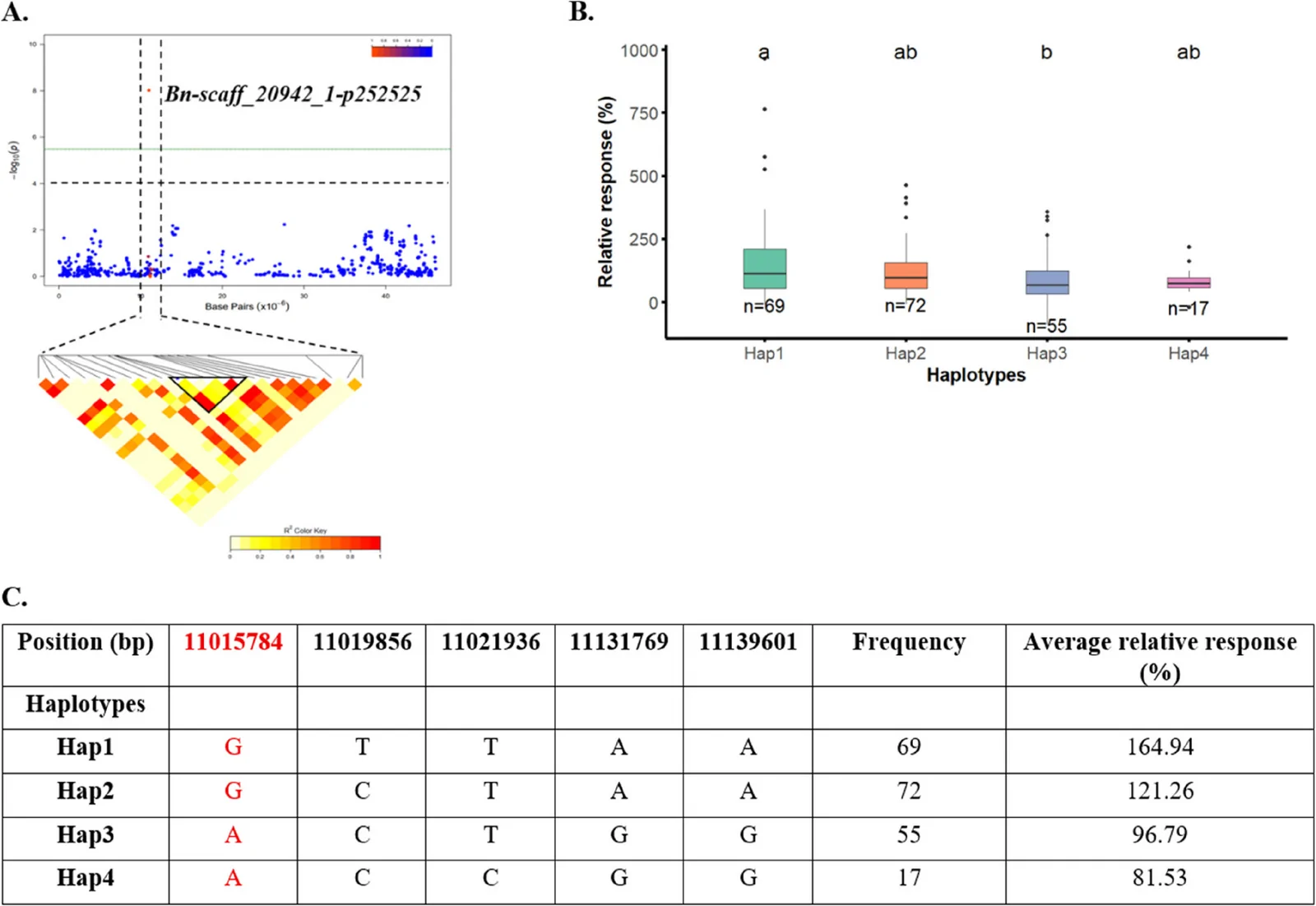
GWAS results and haplotype analysis for ***SNP Bn-scaff_20942_1-p252525*** associated with RR-RE & RR-VI. **A** A Manhattan plot illustrating RR-VI is shown. The green and black horizontal lines mark the Bonferroni & suggestive significance threshold, respectively. Linkage disequilibrium (LD) among SNPs on chromosome C02 is displayed below based on pairwise *r²* values, which are color-coded according to the provided scale. **B** Phenotypic differences in osmopriming response (RR-VI**)** between the identified haplotypes **C** Haplotype analysis of *SNP Bn-scaff_20942_1-p252525* (in red text) and four other linked SNPs. Four haplotypes (Hap1, Hap2, Hap3 and Hap4) were identified in 213 accessions

We identified a key genomic region on chromosome A03, defined by the lead SNP *Bn-A03-p26272560*, to be in strong linkage disequilibrium (LD) with three adjacent SNPs, collectively forming a haplotype block spanning approximately 121 kb region. Haplotype analysis of this block revealed six predominant haplotypes within the association panel, namely: *RR-RE-Hap*1 (TGGA), *RR-RE-Hap*2 (-GGA), *RR-RE-Hap*3 (TATA), *RR-RE-Hap*4 (TATG), *RR-RE-Hap*5 (TAGA), and *RR-RE-Hap*6 (CATG), each exhibiting distinct responses to osmopriming. Among these, *RR-RE-Hap6* (CATG) demonstrated the most substantial priming-induced enhancement, with a mean increase of 44.70%, significantly surpassing *RR-RE-Hap*1 (TGGA, 18.96%), *RR-RE-Hap*3 (TATA, 9.21%), and *RR-RE-Hap*5 (TAGA, 5.88%) (Fig. 7), implicating it as a favourable haplotype for enhanced root performance upon priming. Similarly, on chromosome C02, the significant SNP *Bn-scaff_20942_1-p252525* was in strong LD with four nearby SNPs, forming a second haplotype block spanning approximately 123 kb region. We identified four major haplotypes in this region: *RR-VI-Hap1* (GTTAA), *RR-VI-Hap2* (GCTAA), *RR-VI-Hap3* (ACTGG), and *RR-VI-Hap4* (ACCGG), which also exhibited differential responses to osmopriming. Strikingly, *RR-VI-Hap1* (GTTAA) displayed the most pronounced response, with an average priming-induced increase of 164.94%, considerably higher than *RR-VI-Hap3* (96.79%) and *RR-VI-Hap4* (81.53%) (Fig. 8). These findings highlight both *RR-RE-Hap6* on A03 and *RR-VI-Hap1* on C02 as favourable haplotypes associated with superior osmopriming responsiveness, representing valuable molecular targets for marker-assisted selection aimed at enhancing drought resilience in *Brassica napus* breeding programs.

## Discussion

Root vigour during early seedling establishment is a cornerstone of drought resilience, as rapid root elongation and lateral branching enable seedlings to access soil moisture and nutrients before abiotic stressors intensify [43, 44]. In *Brassica napus* L., weak root systems exacerbate vulnerability to soil compaction and water deficits, leading to uneven stands and yield losses [45]. Seed osmopriming with PEG-6000 mitigates these challenges by synchronizing germination and amplifying root growth kinetics, effectively improving seedling establishment under optimal and suboptimal conditions [9, 46].

Our study provides the first genome-wide association of seed osmopriming responses in *B. napus*, integrating phenotypic and genomic insights to elucidate mechanisms underlying priming-enhanced root vigour. By normalizing primed performance relative to unprimed performance, the relative response (RR) metric allows for the direct assessment of how individual genotypes respond to osmopriming treatment, independent of baseline trait values. This is particularly useful for identifying genetic loci associated with the priming response itself, rather than loci governing constitutive vigour. Our analysis of 228 accessions revealed substantial variation in osmopriming responses for radicle emergence (RR-RE), root length (RR-RL), root growth rate (RR-RGR), and vigour index (RR-VI), with some genotypes showing minimal gains while others exhibited dramatic improvements (Supplementary1, Table S1). The broad spectrum of responses, ranging from minimal to dramatic improvement, underscores the strong genetic component underlying priming efficacy. This finding is critical, as it demonstrates that the potential benefits of priming are not uniform and that genotypic selection will be vital for maximizing its impact. The significant mean improvements we observed in root emergence, elongation, and overall vigour align with established literature demonstrating that priming facilitates metabolic reactivation, leading to accelerated germination and root system development [46–48].

Population structure analysis of 228 spring-type *Brassica napus* L. accessions revealed two distinct genetic subpopulations in which the pronounced genetic subdivision within our panel reflects the documented impact of modern breeding programs on genetic diversity. The relative genetic homogeneity of subpopulation 1 is characteristic of a narrow genetic base, likely resulting from intense selection for elite traits within the DSV breeding program, a phenomenon often leading to genetic erosion of diversity in crops. Conversely, the greater diversity observed within subpopulation 2 can be attributed to the inclusion of IPK gene bank accessions, which represent a broader sample of the species’ genetic potential [33, 49]. This structure necessitates careful interpretation of genetic data, as it may confound association analyses and highlights a potential reliance on a limited gene pool within the elite breeding lines for future improvement.

Linkage disequilibrium (LD) decay differed between the A- and C-subgenomes, reflecting the composite structure of the *B. napus* allopolyploid genome [25]. Rapid LD decay in the A-subgenome suggests higher recombination rates or larger effective population sizes, whereas slower decay in the C-subgenome may result from selective sweeps, reduced recombination, or historical bottlenecks [50]. These patterns have important implications for genome-wide association studies (GWAS) and marker-assisted selection as faster LD in the A-subgenome requires denser markers for effective QTL detection, while slower LD in the C-subgenome allows broader haplotype blocks to be targeted [51]. Understanding population structure and LD dynamics provides a framework for precise genetic mapping and optimized breeding strategies, enabling the exploitation of subgenome-specific variation to accelerate crop improvement in *B. napus*.

**Fig. 9:**
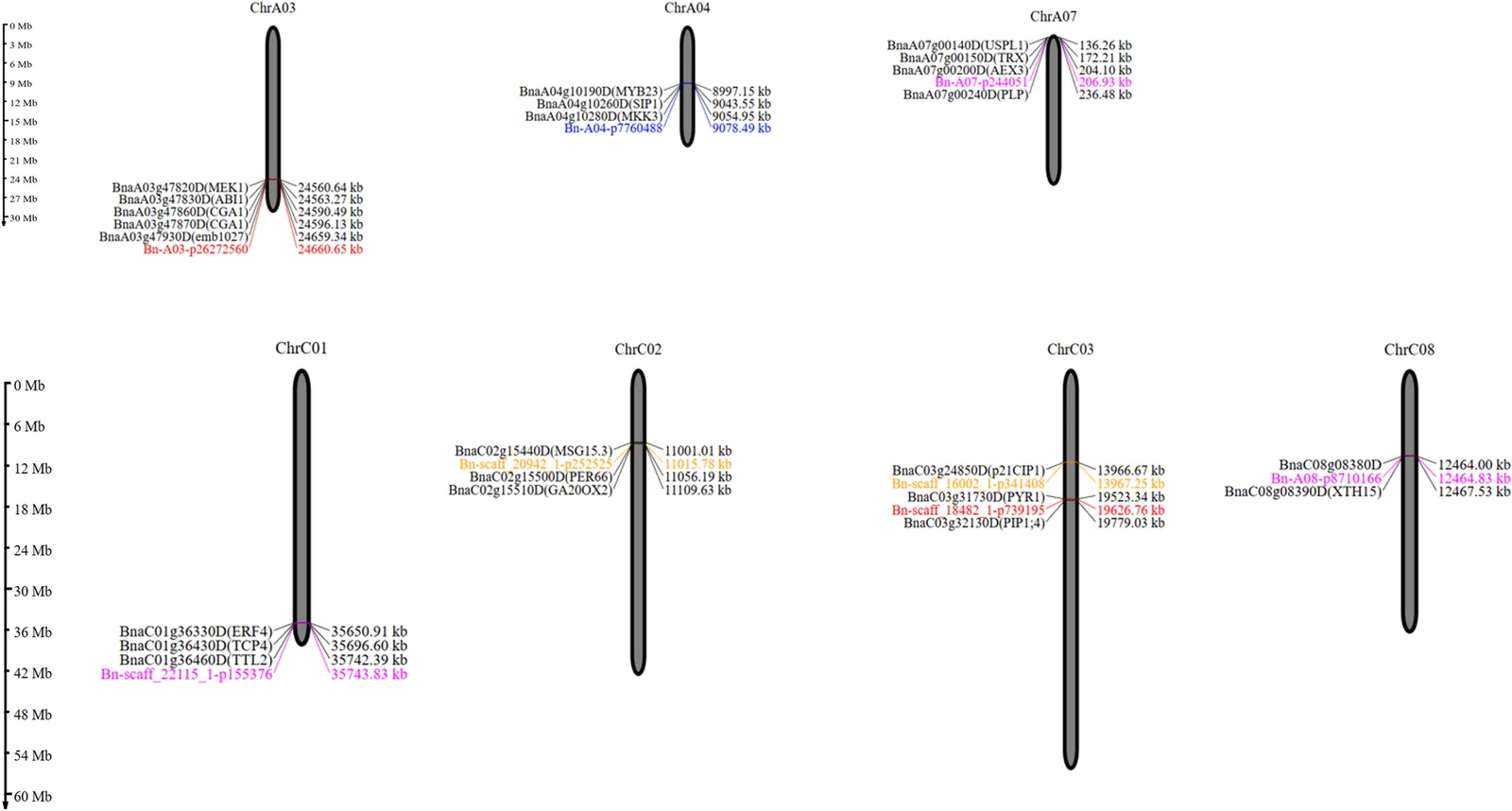
The distribution pattern of key candidate genes and their corresponding SNPs associated with osmopriming response (RR-RE = red, RR-RL = magenta, RR-RGR = blue, RR-RE & RR-VI = orange). Abbreviations of the orthologous genes in *Arabidopsis thaliana* are indicated in brackets following the candidate gene names. Numbers represent the relative physical distances in the genome in kb

Our GWAS identified several significant SNP loci, under our specific osmopriming treatment, that were associated with variation in early root vigour traits. These SNPs co-localized with candidate genes involved in hormonal crosstalk, water dynamics, cell wall remodelling, root development, and redox homeostasis, offering a plausible mechanistic framework for the observed root improvement (Fig. 9; Table 3). While some traits showed extremely high correlations, the overlap of associated loci was not as extensive as expected. This discrepancy may arise because correlations could be driven by polygenic effects involving many shared loci of small effect that fall below the significance threshold in this study, as well as by linkage disequilibrium patterns and limited statistical power. Nonetheless, the significant associations detected in our study highlight key candidate genes.

Among the associations detected, one of the most notable was for SNP *Bn-A03-p26272560*, located near the gene *ABI1* (*BnaA03g47830D*), a member of the clade A *PP2C* phosphatase family known to function as a negative regulator of abscisic acid (ABA) signaling [52]. Under non-stress conditions, ABA receptors (*PYR/PYL/RCAR*) are inactive [53]. Upon ABA accumulation during stress or dormancy, ABA binds to its receptors, which then sequester and inhibit PP2Cs like *ABI1*. This inhibition relieves the constitutive repression that *ABI1* imposes on downstream *SnRK2* kinases. Active *SnRK2s* then phosphorylate numerous targets, including transcription factors like *ABFs*, to activate stress-responsive genes and suppress premature germination [52, 53]. Therefore, natural variation in the vicinity around *ABI1* locus may likely modulate the sensitivity of the entire ABA signaling cascade. Recent findings have demonstrated the functional significance of this pathway in seed priming, revealing that the chemical inhibition of ABA signaling via a receptor antagonist markedly enhances seed germination under high-temperature stress and significantly boosts the effectiveness of osmopriming in lettuce [54]. *ABI1* is thus a high-priority candidate for marker-assisted selection to breed for reduced ABA sensitivity and enhanced osmopriming response during seedling establishment. Furthermore, *emb1027* (*BnaA03g47930D*), which contains the SNP *Bn-A03-p26272560* in its exonic region and encodes an arginyl-tRNA synthetase, suggests a potential role in metabolic priming through enhanced mitochondrial translation, aligning with reports linking ATP biosynthesis to successful seed imbibition [16, 55]. In proximity, *CGA1* (*BnaA03g47860D/BnaA03g47870D*), a cytokinin-responsive transcription factor, may enhance abiotic stress adaptation and chloroplast development post-priming [56].

Another key finding from our GWAS analysis highlights the essential role of controlled water uptake. The *PIP1;4* (*BnaC03g32130D*), which encodes a plasma membrane intrinsic aquaporin protein, is located in the LD region of SNP *Bn-scaff_18482_1-p739195*, associated with RR-RE. *PIP* aquaporins facilitate the passive transport of water across membranes and are instrumental in cell expansion and seedling development [57, 58]. A favourable *PIP1;4* allele may likely enable more rapid and controlled rehydration during the critical imbibition phase, directly translating to more synchronous radicle emergence. This is consistent with earlier studies indicating that osmopriming induces the upregulation of *PIP1;4* genes in *B. napus*, thereby facilitating more efficient water uptake, an essential factor for seed germination and early root establishment [59]. Similarly, *Bn-A04-p7760488*, associated with RR-RGR, is found near *BnaA04g10260D*, homologous to *seed imbibition 1-like* (*SIP1*), a gene implicated in sugar transport, drought tolerance, and regulating water uptake during seed imbibition [60]. The identification of *PIP1;4* and *SIP1* within priming-responsive loci underscores the importance of tightly controlled water absorption during the rehydration phase, which is crucial for activating metabolic pathways that lead to improved seedling root vigour upon priming. The SNP *Bn-scaff_20942_1-p252525*, associated with both RR-RE and RR-VI, exemplifies hormonal crosstalk in priming response. This SNP is located in close proximity to *GA20ox2* (*BnaC02g15510D*), a gene encoding a key gibberellin 20-oxidase enzyme critical for gibberellin biosynthesis, a pathway that centrally regulates plant growth and development [61]. GA is a potent antagonist of ABA; its biosynthesis is essential for initiating the metabolic processes required for radicle protrusion and subsequent root elongation [62]. The presence of *GA20ox2* (*BnaC02g15510D*) within priming-responsive loci indicates that gibberellin-driven root elongation may counter ABA-mediated growth suppression, aligning with Nakaune et al. (2012) who found the upregulation of *GA20ox* genes in NaCl-primed tomato seeds [63]. Seed imbibition also marks a critical metabolic transition, rapidly re-activating mitochondrial oxidative phosphorylation and triggering a spatially regulated burst of reactive oxygen species (ROS) in the embryo [64, 65]. This ROS production operates within a narrow oxidative window, where it functions as a crucial signaling cue to promote dormancy release and germination through hormonal cross-talk and cell wall modification [66, 67]. However, excessive ROS accumulation leads to oxidative damage, necessitating precise redox control [68, 69]. Co-mapped in the priming-responsive loci are *MSG15.3* (*BnaC02g15440D*) belonging to the GMC oxidoreductase family and *PER66* (*BnaC02g15500D*) belonging to the peroxidase family, which further underscore redox regulation as a key component of priming efficacy [46, 70]. These roles are reinforced by *GRXS12* (*BnaA07g00150D*), a thioredoxin superfamily protein that contributes to redox buffering during seed germination, and *TTL2* (*BnaC01g36460D*), which has the SNP *Bn-scaff_22115_1-p155376* in its exonic region and is known to stabilize proteins under oxidative stress [70]. These assumptions align slightly with the findings of Kubala et al. (2015), who reported the differential expression of *MSG15.3* gene in *B. napus* following osmopriming treatment [46].

The SNP *Bn-A08-p8710166* on chromosome C08, associated with RR-RL, harbours *XTH15* (*BnaC08g08390D*) within its linkage disequilibrium window. Consistent with the oxidative remodeling hypothesis, *XTH15* mediates xyloglucan modification, facilitating cell wall loosening and promoting root elongation under osmotic stress [71]. *XTH* gene has also been previously implicated in seed priming responses, underscoring its pivotal role in osmopriming-induced root growth enhancement [72].

Additional structural and signaling elements, including *TCP4* (*BnaC01g36430D*), *ERF4* (*BnaC01g36330D*), and *PLP*-dependent enzymes (*BnaA07g00240D*), underscore the intricate regulatory networks underpinning transcriptional and metabolic priming.

The association on chromosome C01, tagged by SNP *Bn-scaff_22115_1-p155376*, is particularly compelling as it overlaps a major QTL hotspot previously reported to be associated with seed number and seed weight [73, 74]. This region, spanning approximately 35.27 to 36.27 Mb, contains the QTLs *DH-spsl*, *DH-tsm*, *S/SilN11-1* and *TkwN11-1*, which were identified in biparental populations derived from Express617 × V8 and Express617 × R53, respectively [75]. Interestingly, the transcription factor gene *TCP4* (*BnaC01g36430D*), regulated by *miR319* and affects root growth is positioned within these intervals and emerges as a high-confidence candidate gene underlying both seed quality and priming-responsive QTLs [76]. This co-location suggests a shared genetic architecture, in which pleiotropic genes that regulate fundamental processes in early seed development and composition also modulate the seed’s responsiveness to pre-germination treatments such as osmopriming. In addition, our analysis also identified that the priming-responsive locus on chromosome C03, tagged by the SNP *Bn-scaff_16002_1-p341408*, was in close proximity to a previously reported seed yield (SY) QTL [73]. This SY QTL, which spans the physical distance of 10.03 to 11.03 Mb on chromosome C03, encompasses the yield-related loci *DH-y06*, *MPH-y06*, and *GyN13-1*, which were identified in biparental populations of Express617 × V8 and Express617 × R53 [75]. The midpoints of the priming locus and the SY QTL are separated by approximately 3.5 Mb, suggesting a potential genomic relationship between priming responsiveness and seed yield architecture. Another intriguing overlap exists between our study and the work of Yang et al. (2024), who identified a locus for total primary root length on chromosome A07 [77]. Their candidate gene, *BnaA07g00160D* (a nucleotide sugar transporter), and lead SNP, *Bn_A07_p3921656* are located near our priming-responsive locus for RR-RL, tagged by SNP *Bn-A07-p244051*, which is situated within intron 8 of *AEX-3* gene (*BnaA07g00200D*). The *AEX-3* gene encoding the MAP kinase-activating death domain (MADD) protein, functions as a guanine nucleotide exchange factor (GEF) that regulates *Rab3* and *Rab27* GTPases and is implicated in vesicle trafficking and exocytosis processes critical for cell expansion and root growth [78]. The overlap of these loci suggests a potential genomic region on chromosome A07 that may play a fundamental role in regulating root elongation mechanisms, both under standard conditions and in response to priming. This convergence highlights a promising candidate for future functional validation to elucidate its role in root developmental plasticity. We found no other overlap between the confidence intervals of the significant SNPs reported in this study and any other existing QTLs.

Our haplotype analysis revealed two key haplotype blocks associated with differential osmopriming responses. The chromosome A03 block (lead SNP *Bn-A03-p26272560*) contained six haplotypes, with *RR-RE-Hap6* (CATG) showing the strongest priming-induced enhancement. This block encompasses multiple functionally relevant genes, such as *MKK1*, *ABI1*, *CGA1*, and *emb1027*, suggesting that the superior performance of *RR-RE-Hap6* is likely the cumulative effect of several linked genes rather than a single causal gene. Similarly, the chromosome C02 block (lead SNP *Bn-scaff_20942_1-p252525*) contained four haplotypes, with *RR-VI-Hap1* (GTTAA) exhibiting the most pronounced response. This block includes genes such as *TCP19*, *PER66*, and *GA20ox2*, supporting the idea that the haplotype effect likely arises from the combined influence of multiple linked loci. Collectively, these findings indicate that these haplotype blocks capture key genomic regions controlling osmopriming-induced early root vigour and may serve as robust functional markers for marker-assisted breeding focused on improving seedling establishment.

While our study provides valuable insights into the genetic basis of osmopriming response affecting early root vigour traits through genome-wide association studies (GWAS) in a diverse *Brassica napus* accession panel, a few limitations have also been identified. Firstly, the moderate size of the population may restrict the power to detect rare alleles and loci with smaller effect sizes, a common constraint in crop GWAS. Secondly, the phenotyping was conducted under a single environmental condition, which restricts our understanding of genotype-by-environment interactions and limits the generalisability of the findings to other osmotic or moisture regimes. Furthermore, the stability of the identified SNPs across different environments and genetic backgrounds was not assessed, creating uncertainty regarding their consistency and utility in broader breeding contexts. Different candidate genes were identified; however, functional validation of the genes remains to be performed. Techniques such as mutant analysis, RT-qPCR, or RNA-seq can assist in confirming the expression or causal role in the response of these candidate genes. Therefore, while this work lays a critical foundation for understanding the genetic architecture of osmopriming response in oilseed rape, further multi-environment trials, utilising larger and more diverse populations, and functional genomic analyses are essential to substantiate these candidate loci and elucidate their precise regulatory mechanisms, ultimately facilitating their application in crop improvement programmes [79].

## Conclusion

In conclusion, our results indicate that enhancement of early root vigour traits through osmopriming is associated with coordinated genetic networks, highlighting allelic variants that regulate priming-adaptive developmental responses. In this study, we identified several such variants linked to variation in osmopriming response, offering new insights into the genetic basis of priming-induced phenotypic plasticity. Functional annotation revealed involvement of abscisic acid signaling, kinase activities, redox regulation, water uptake, and cell wall remodeling which collectively contribute to the modulation of root development under stress conditions (Supplementary1, Table S7; Supplementary2, Fig. S6). Identifying loci associated with osmopriming response will enable the development of molecular markers that can guide marker-assisted and genomic selection for improving early seedling root vigour in *Brassica napus*. Targeting alleles associated with enhanced osmopriming response at 72 h after sowing may enhance early germination rate and root elongation, which reflects very early root vigour. While these loci are more indicative of initial germination dynamics than sustained growth or long-term survival, such early advantages could still contribute to later-stage establishment under variable moisture conditions, even though this link remains to be validated over longer developmental time scales. These insights provide a foundation for exploiting beneficial variation in germplasm and accelerating the breeding of resilient cultivars with superior early growth and performance. By integrating SNP- and haplotype-level analyses, this work establishes a strong foundation for identifying functional variants and facilitating the development of cultivars with improved early root vigour through genomics-assisted breeding.

## Supplementary Information

Supplementary material 1.

Supplementary material 2.

## Data Availability

All data generated or analysed during this study are included in this published article [and its supplementary information files] or are available from the corresponding author upon reasonable request.

## Abbreviations

GWAS: Genome-wide association study
SNP: Single nucleotide polymorphism
BLINK: Bayesian-information and linkage-disequilibrium iteratively nested keyway
CV: Coefficient of variation
FDR: False discovery rate
H^2^: Broad sense heritability
RR: Relative response
RE: Radicle emergence
RL: Root length
RGR: Root growth rate
VI: Vigour index
LD: Linkage disequilibrium
QTL: Quantitative trait loci
MAF: Minor allele frequency
MAS: Marker-assisted selection
MPa: Megapascal
Mb: Megabase
kb: Kilobase
