# Correction: Genome-wide association mapping reveals genetic loci underlying phenotypic variation in early root vigour improvement induced by osmopriming in *Brassica napus* L

**DOI:** 10.1186/s12870-026-09985-7

**Published:** 2026-10-15

**Authors:** Kingsley Onyinye Ibeabuchi, Maira Marins Dourado, Stefan Scholten, Ulf Feuerstein

**Affiliations:** 1 Division of Crop Plant Genetics, Department of Crop Sciences, GeorgAugust- University of Göttingen, Göttingen, Germany; 2 Department of Seed Technology, Deutsche Saatveredelung, Asendorf, Germany; 3 Faculty of Science, Swammerdam Institute for Life Sciences, University of Amsterdam, Amsterdam, Netherlands; 4 Centre for Integrated Breeding Research, Georg-August-University of Göttingen, Göttingen, Germany

**Correction: BMC Plant Biol 25, 1460 (2025)**

**https://doi.org/10.1186/s12870-025-07540-4**

Following publication of the original article, the author identified error in the equation on page 5 where the “+” sign between Rₖ and ϵ_i_ⱼₖ was inadvertently omitted.

The equation previously read:

$$Y\;ijk=\mu+Ti+Gj+\left(T\times G\right)ij+Rk\varepsilon ijk$$

The corrected equation reads:

$$Y\;ijk=\mu+Ti+Gj+\left(T\times G\right)ij+Rk+\varepsilon ijk$$

The original article [1] has been updated.
