# Supplementary material for: Genome-wide association mapping reveals genetic loci underlying phenotypic variation in early root vigour improvement induced by osmopriming in Brassica napus L
Source: BMC Plant Biol. 2025 Oct 29;25:1460. doi: 10.1186/s12870-025-07540-4 (PMC12570839; doi:10.1186/s12870-025-07540-4)
Supplement: Supplementary file 2 — Supplementary material 2. [file 12870_2025_7540_MOESM2_ESM.docx]

**Supplementary material 2 for:**

**Genome-wide association mapping reveals genetic loci underlying phenotypic variation in early root vigour improvement induced by osmopriming in *Brassica napus* L.**

**Authors:** Kingsley Onyinye Ibeabuchi^1,2*^, Maira Marins Dourado^3^, Stefan Scholten^1,4^,Ulf Feuerstein^2^

*^1^Georg-August-University of Göttingen, Göttingen, Germany, Division of Crop Plant Genetics, Department of Crop Sciences*

*^2^Deutsche Saatveredelung AG, Asendorf, Germany, Department of Seed Technology*

*^3^University of Amsterdam, Amsterdam, Netherlands, Faculty of Science, Swammerdam Institute for Life Sciences*

*^4^Georg-August-University of Göttingen, Göttingen, Germany, Centre for Integrated Breeding Research*

*Correspondence: [kingsley-onyinye.ibeabuchi@agr.uni-goettingen.de](mailto:kingsley-onyinye.ibeabuchi@agr.uni-goettingen.de)


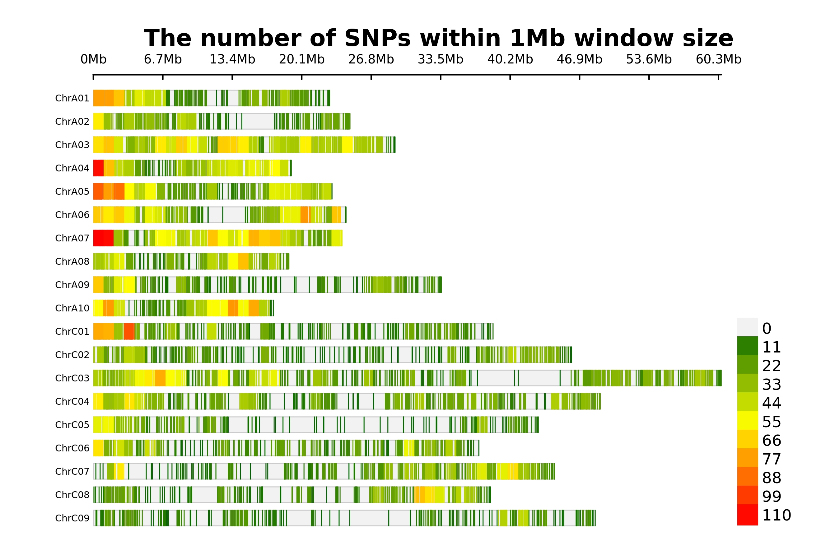


**Figure S1.** Chromosome-wise SNP density plot illustrating the number of SNPs within 1 Mb windows. The horizontal axis represents chromosome length in megabases (Mb), and variations in colour indicate SNP density levels


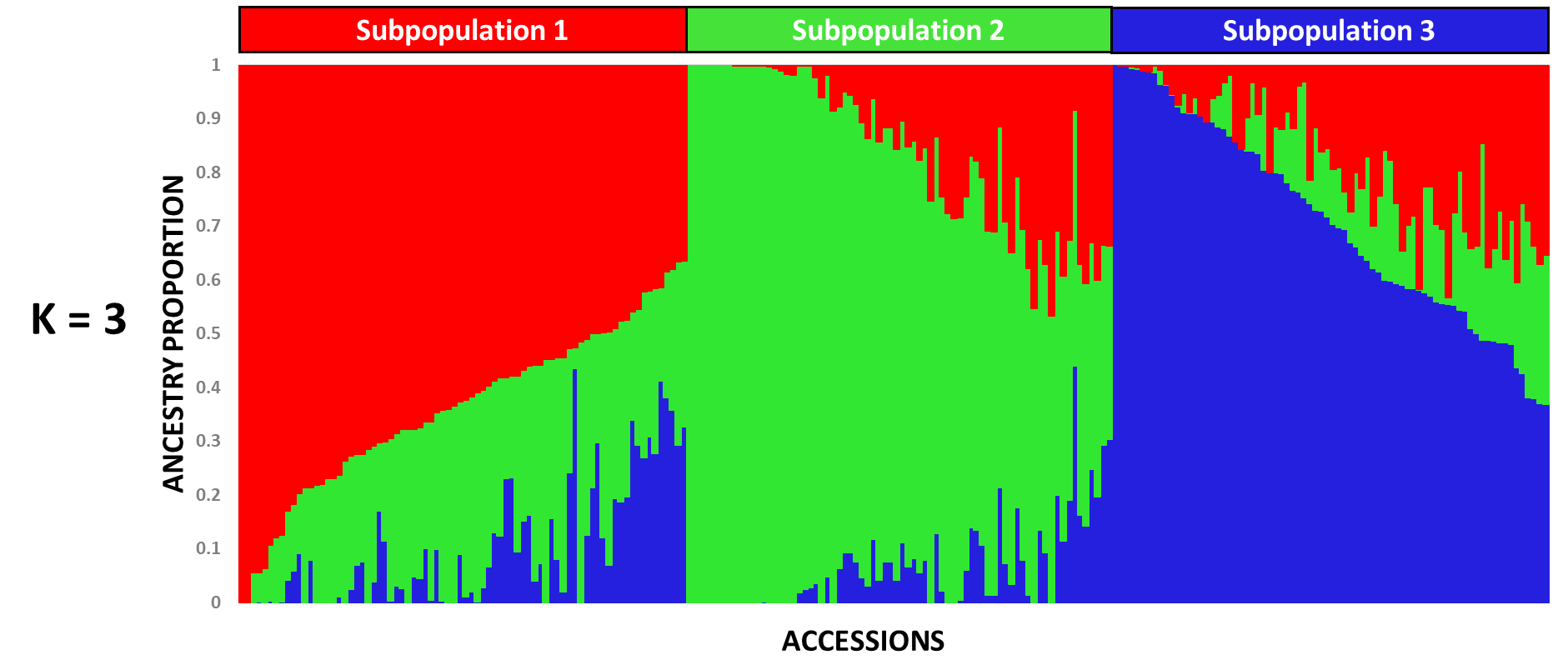


**Figure S2.** Estimated ancestry proportions of accessions at K = 3 based on STRUCTURE analysis


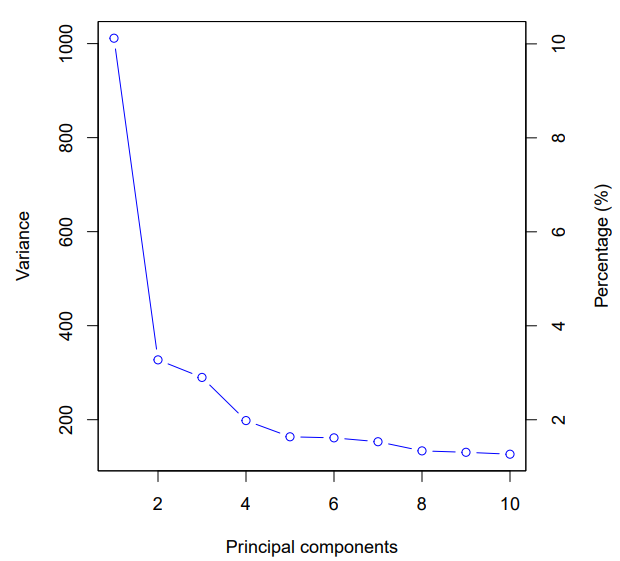


**Figure S3.** Scree plot showing the proportion of genetic variance explained by principal components derived from SNP-based PCA in the GWAS panel


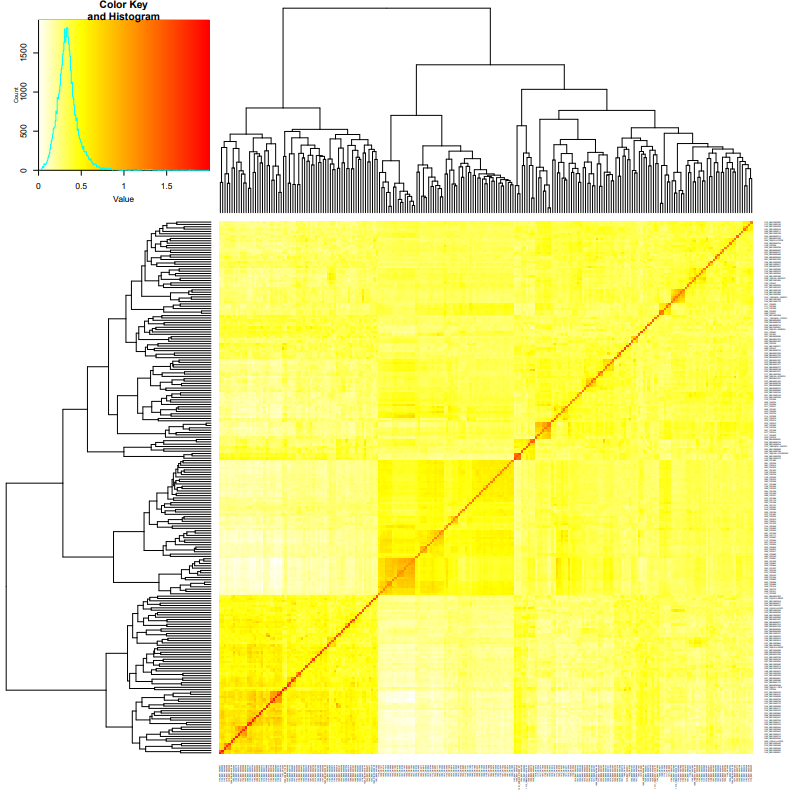


**Figure S4.** Heatmap representation of the kinship matrix illustrating genetic relatedness across the population. Darker regions indicate stronger genetic similarity between individual genotypes.

ChrA07 - Bn-A07-p244051 ChrC01 - Bn-scaff_22115_1-p155376


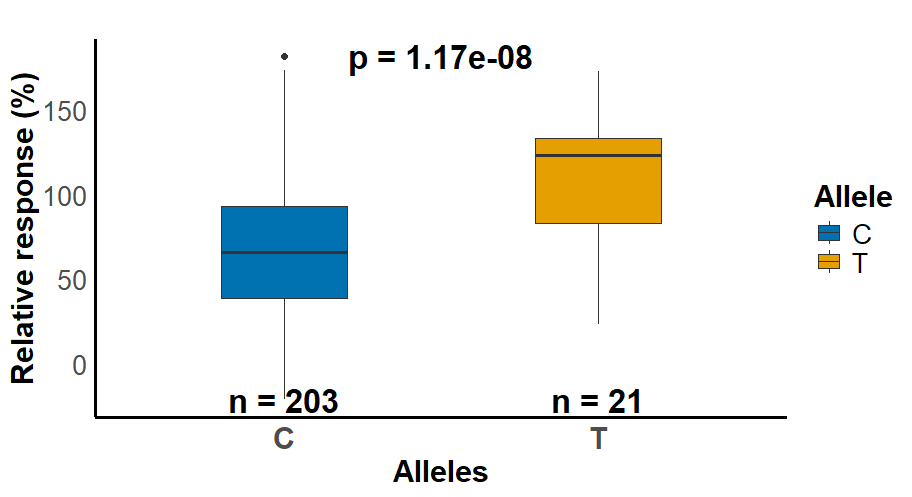

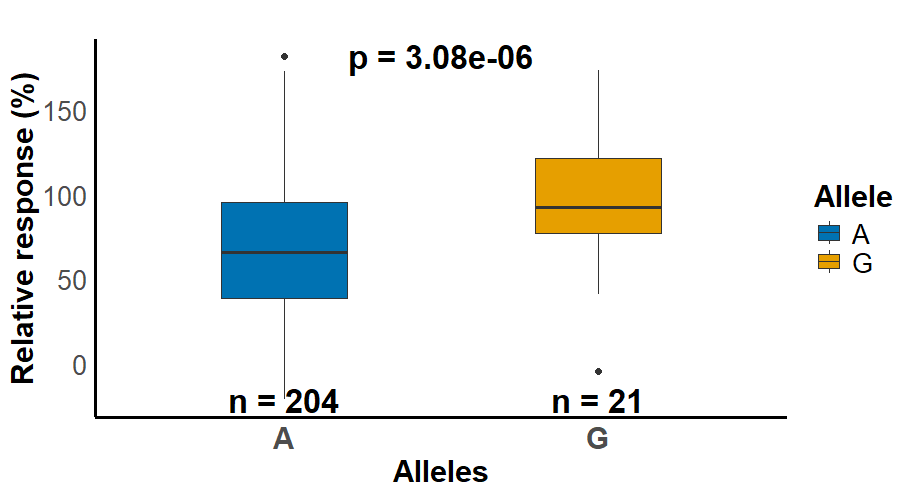


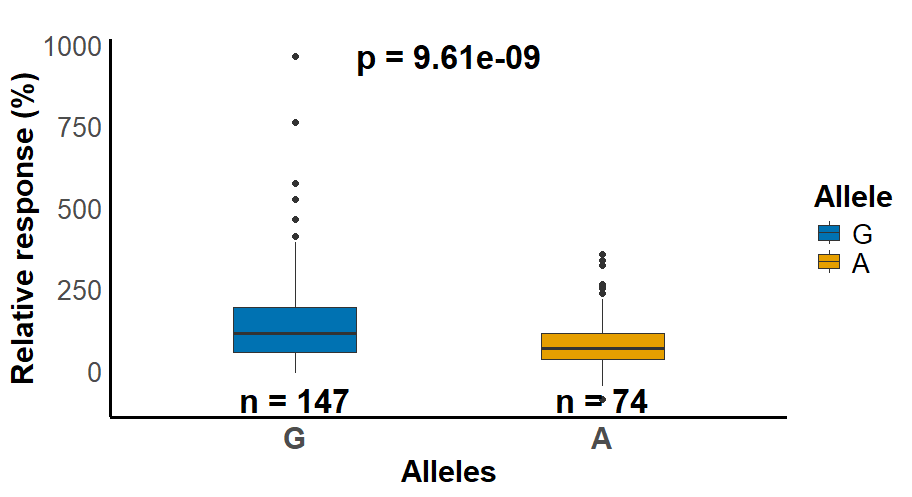

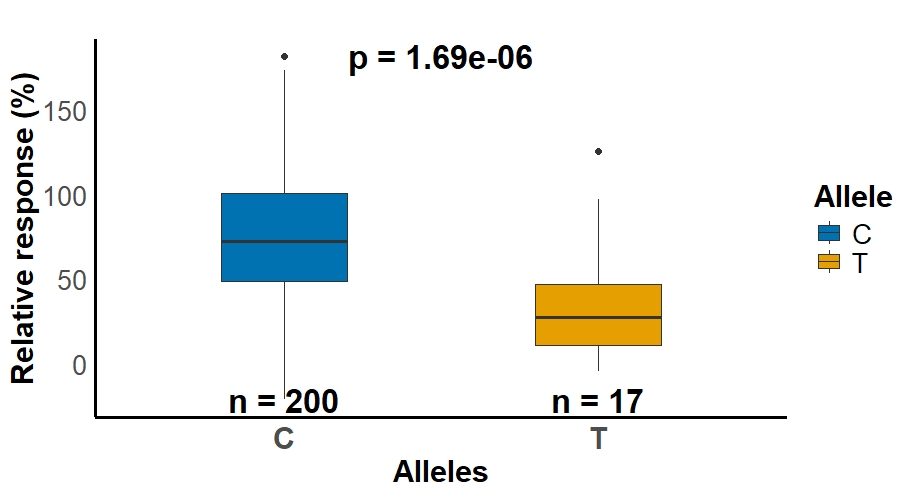
ChrC08 - Bn-C08-p8710166 ChrC02 - Bn-scaff_20942_1-p252525

**Figure S5.** Boxplots of the allelic effects of four SNPs that exceeded the genome-wide significant threshold (-log10 p ≥ 5.48) associated with osmopriming response


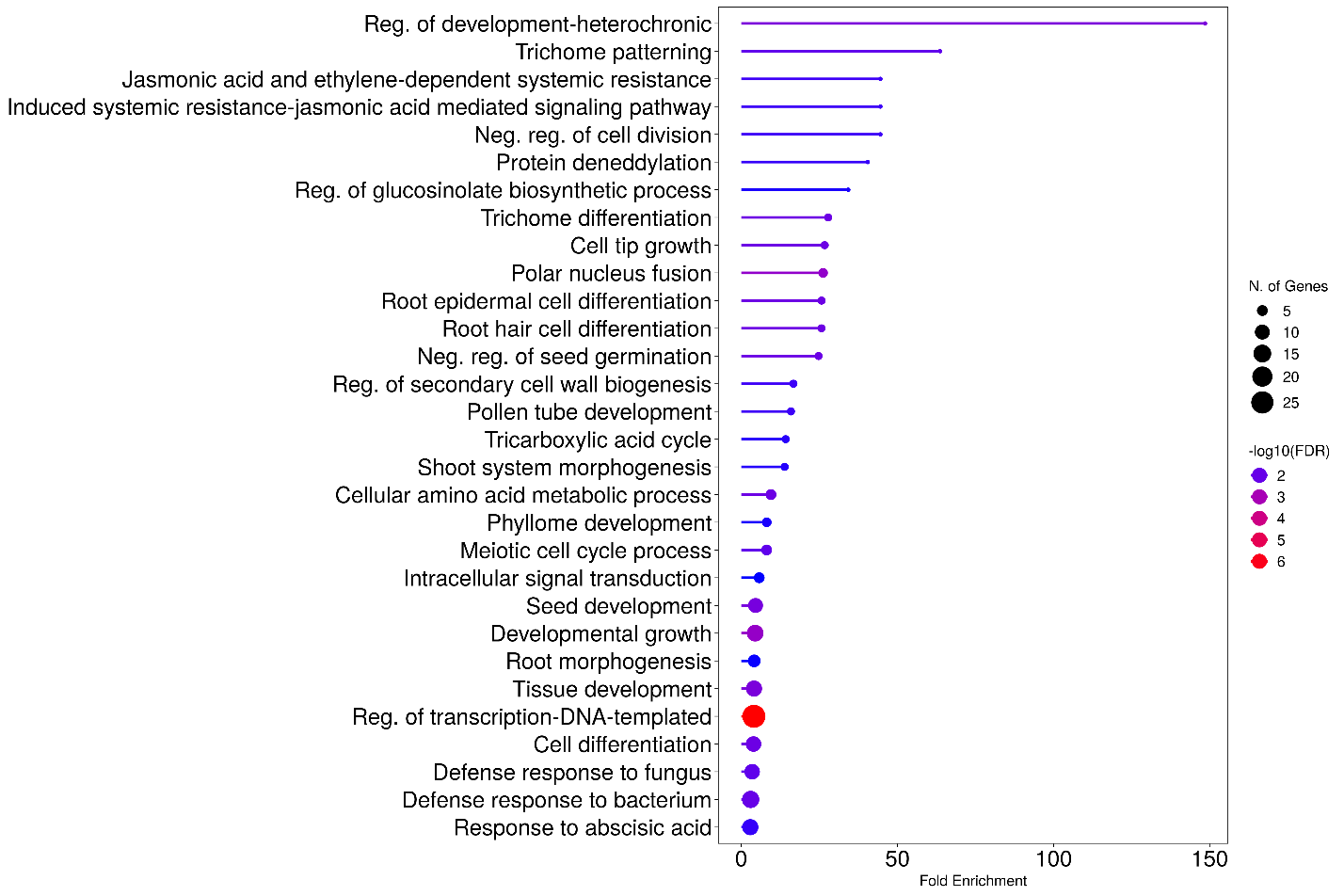


**Figure S6.** Significantly enriched Gene Ontology (GO) terms (FDR < 0.05) for candidate genes within priming-responsive loci, as identified by the GWAS BLINK model.
